# Supplementary material for: Coexistence from a lion’s perspective: Movements and habitat selection by African lions (Panthera leo) across a multi-use landscape
Source: PLoS One. 2024 Oct 3;19(10):e0311178. doi: 10.1371/journal.pone.0311178 (PMC11449311; doi:10.1371/journal.pone.0311178)
Supplement: S1 Table — (DOCX) [file pone.0311178.s001.docx]

| S1 Table. Collared Lions: categories, collaring duration (months), and proportional representation by all lions and by its category | | | | | | | |
| --- | --- | --- | --- | --- | --- | --- | --- |
| Lion ID | Sex | Collared months | Nomadic months | % of all positions | % of Females | % of Resident Males | % of Nomadic Males |
| Nadine | F | 56.4 | NA | 13% | 30% |  |  |
| Nayomi | F | 33.9 | NA | 8% | 18% |  |  |
| LK112 | F | 12.4 | NA | 3% | 7% |  |  |
| MG2-C | F | 3.4 | NA | 1% | 2% |  |  |
| MG74 | F | 6.2 | NA | 1% | 3% |  |  |
| LK124 | F | 1.7 | NA | 0% | 1% |  |  |
| LK107 | F | 25.2 | NA | 6% | 13% |  |  |
| MAS-8 | F | 50.3 | NA | 11% | 27% |  |  |
| Bahati | M | 9.8 | 0 | 2% |  | 7% | 0% |
| Kalamas | M | 10.8 | 10.8 | 2% |  | 0% | 9% |
| Kijana | M | 3.7 | 0 | 1% |  | 3% | 0% |
| MAS-9 | M | 3.5 | 3.5 | 1% |  | 0% | 3% |
| TWH-2 | M | 48 | 17.3 | 11% |  | 23% | 15% |
| MG127 | M | 1.7 | 0 | 0% |  | 1% | 0% |
| MG130 | M | 41.1 | 0 | 9% |  | 31% | 0% |
| TWH-32 | M | 7.9 | 7.9 | 2% |  | 0% | 7% |
| LK140 | M | 26.7 | 11.6 | 6% |  | 11% | 10% |
| MAS-11 | M | 10.8 | 10.8 | 2% |  | 0% | 9% |
| MAS-13 | M | 31.5 | 31.5 | 7% |  | 0% | 27% |
| Orbili | M | 6.7 | 6.7 | 2% |  | 0% | 6% |
| Puyol | M | 20.6 | 0 | 5% |  | 16% | 0% |
| THI-A | M | 25.7 | 16.6 | 6% |  | 7% | 14% |
| Total |  | 437.7 | 116.5 |  |  |  |  |
