# Supplementary material for: Coexistence from a lion’s perspective: Movements and habitat selection by African lions (Panthera leo) across a multi-use landscape
Source: PLoS One. 2024 Oct 3;19(10):e0311178. doi: 10.1371/journal.pone.0311178 (PMC11449311; doi:10.1371/journal.pone.0311178)
Supplement: S2 Table — (DOCX) [file pone.0311178.s002.docx]

| **S2 Table.** Test of the best radius over which to calculate the intensity of human activity on the landscape (i.e., boma density) for each lion category in Ngorongoro Conservation Area, Tanzania. | | | |
| --- | --- | --- | --- |
| Radius | AIC | $\Delta$AIC | Type |
| 1km | 1122878.5 | 0.0 | Male (nomadic) |
| 5km | 1122937.4 | 58.8 | Male (nomadic) |
| 3km | 1122968.4 | 89.8 | Male (nomadic) |
| 1km | 690559.8 | 0.0 | Male (resident) |
| 3km | 690596.3 | 36.5 | Male (resident) |
| 5km | 690656.7 | 96.9 | Male (resident) |
| 5km | 1478401.6 | 0.0 | Female |
| 3km | 1478420.1 | 18.5 | Female |
| 1km | 1478453.4 | 51.9 | Female |
