# Supplementary material for: Coexistence from a lion’s perspective: Movements and habitat selection by African lions (Panthera leo) across a multi-use landscape
Source: PLoS One. 2024 Oct 3;19(10):e0311178. doi: 10.1371/journal.pone.0311178 (PMC11449311; doi:10.1371/journal.pone.0311178)
Supplement: S3 Table — The dry season is the reference level. (DOCX) [file pone.0311178.s003.docx]

| **S3 Table.** Parameters estimated for the difference in mean squared displacement (MSD) of lion movements between wet and dry seasons. The dry season is the reference level. | | | | | |
| --- | --- | --- | --- | --- | --- |
| Type | Intercept | SE.Intercept | Slope.Coefficient | SE.Slope | p.value |
| Male (Nomadic) | 10.55577 | 0.00003 | 0.57234 | 0.00004 | <0.0001 |
| Male (Resident) | 9.88698 | 0.00004 | -0.13740 | 0.00006 | <0.0001 |
| Female | 8.14220 | 0.00007 | 0.13676 | 0.00010 | <0.0001 |
