# Supplementary material for: Coexistence from a lion’s perspective: Movements and habitat selection by African lions (Panthera leo) across a multi-use landscape
Source: PLoS One. 2024 Oct 3;19(10):e0311178. doi: 10.1371/journal.pone.0311178 (PMC11449311; doi:10.1371/journal.pone.0311178)
Supplement: S4 Table — Dens_human represents intensity of human activity while Dist_human represents distance to human activity. (DOCX) [file pone.0311178.s004.docx]

| **S4 Table.** Model selection results for step-selection functions for female, resident male, and nomadic male lions in Ngorongoro Conservation Area, Tanzania between 2012-2023. Dens_human represents intensity of human activity while Dist_human represents distance to human activity. | | | |
| --- | --- | --- | --- |
| Lion Type | Model | AIC | DeltaAIC |
| Females | ~Cover+Dens_human+Dist_river+Dist_human+EVI+TRI+Cover*Dist_human+ Cover*Dens_human | 1477521 | 0 |
| Females | ~Cover+Dens_human+Dist_river+Dist_human+EVI+TRI | 1477956 | 434.6 |
| Females | ~Cover+Dist_river+EVI+TRI | 1477984 | 462.8 |
| Females | ~Cover | 1478475 | 954.3 |
| Females | ~Dist_river+EVI+TRI | 1483147 | 5625.6 |
| Females | ~Dens_human+Dist_human | 1483948 | 6426.6 |
| Males (resident) | ~Cover+Dens_human+Dist_river+Dist_human+EVI+TRI+Cover*Dist_human+ Cover*Dens_human | 691869 | 0 |
| Males (resident) | ~Cover+Dens_human+Dist_river+Dist_human+EVI+TRI | 691947 | 77.9 |
| Males (resident) | ~Cover+Dist_river+EVI+TRI | 692140 | 270.8 |
| Males (resident) | ~Cover | 692431 | 562 |
| Males (resident) | ~Dist_river+EVI+TRI | 693517 | 1647.9 |
| Males (resident) | ~Dens_human+Dist_human | 693727 | 1857.8 |
| Males (nomadic) | ~Cover+Dens_human+Dist_river+Dist_human+EVI+TRI+Cover*Dist_human+ Cover*Dens_human | 1122534 | 0 |
| Males (nomadic) | ~Cover+Dens_human+Dist_river+Dist_human+EVI+TRI | 1122645 | 111.1 |
| Males (nomadic) | ~Cover+Dist_river+EVI+TRI | 1122769 | 234.6 |
| Males (nomadic) | ~Cover | 1122971 | 436.7 |
| Males (nomadic) | ~Dist_river+EVI+TRI | 1124072 | 1537.9 |
| Males (nomadic) | ~Dens_human+Dist_human | 1124148 | 1613.6 |
|  |  |  |  |
