# Supplementary material for: Coexistence from a lion’s perspective: Movements and habitat selection by African lions (Panthera leo) across a multi-use landscape
Source: PLoS One. 2024 Oct 3;19(10):e0311178. doi: 10.1371/journal.pone.0311178 (PMC11449311; doi:10.1371/journal.pone.0311178)
Supplement: S1 Fig — Livestock attacks were reported regularly by local communities. When reports were received, locally trained field staff, with close ties to the pastoralist communities reporting the attacks, performed field visits to verify the attack and the type of predator involved. (DOCX) [file pone.0311178.s005.docx]

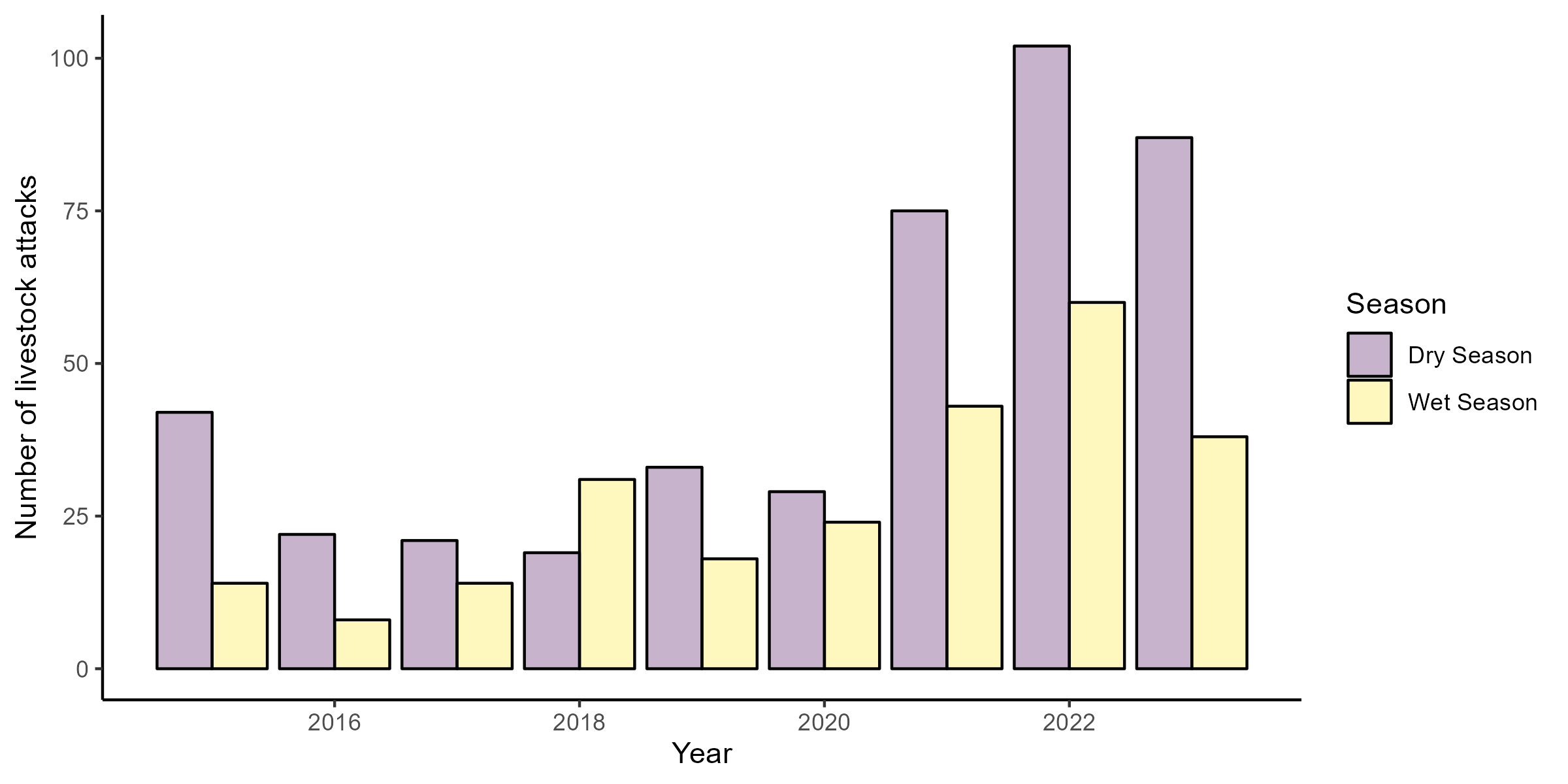


**S1 Figure.** Total number of attacks by lions on livestock in the study area (Fig 1) from 2015-2024, split between wet and dry-season attacks. Livestock attacks were reported regularly by local communities. When reports were received, locally trained field staff, with close ties to the pastoralist communities reporting the attacks, performed field visits to verify the attack and the type of predator involved.
