# Supplementary material for: Coexistence from a lion’s perspective: Movements and habitat selection by African lions (Panthera leo) across a multi-use landscape
Source: PLoS One. 2024 Oct 3;19(10):e0311178. doi: 10.1371/journal.pone.0311178 (PMC11449311; doi:10.1371/journal.pone.0311178)
Supplement: S2 Fig — Study area in the Ngorongoro Conservation Area showing the distribution of rivers (A) and bomas (B). The green polygon is the Ngorongoro Crater. (DOCX) [file pone.0311178.s006.docx]

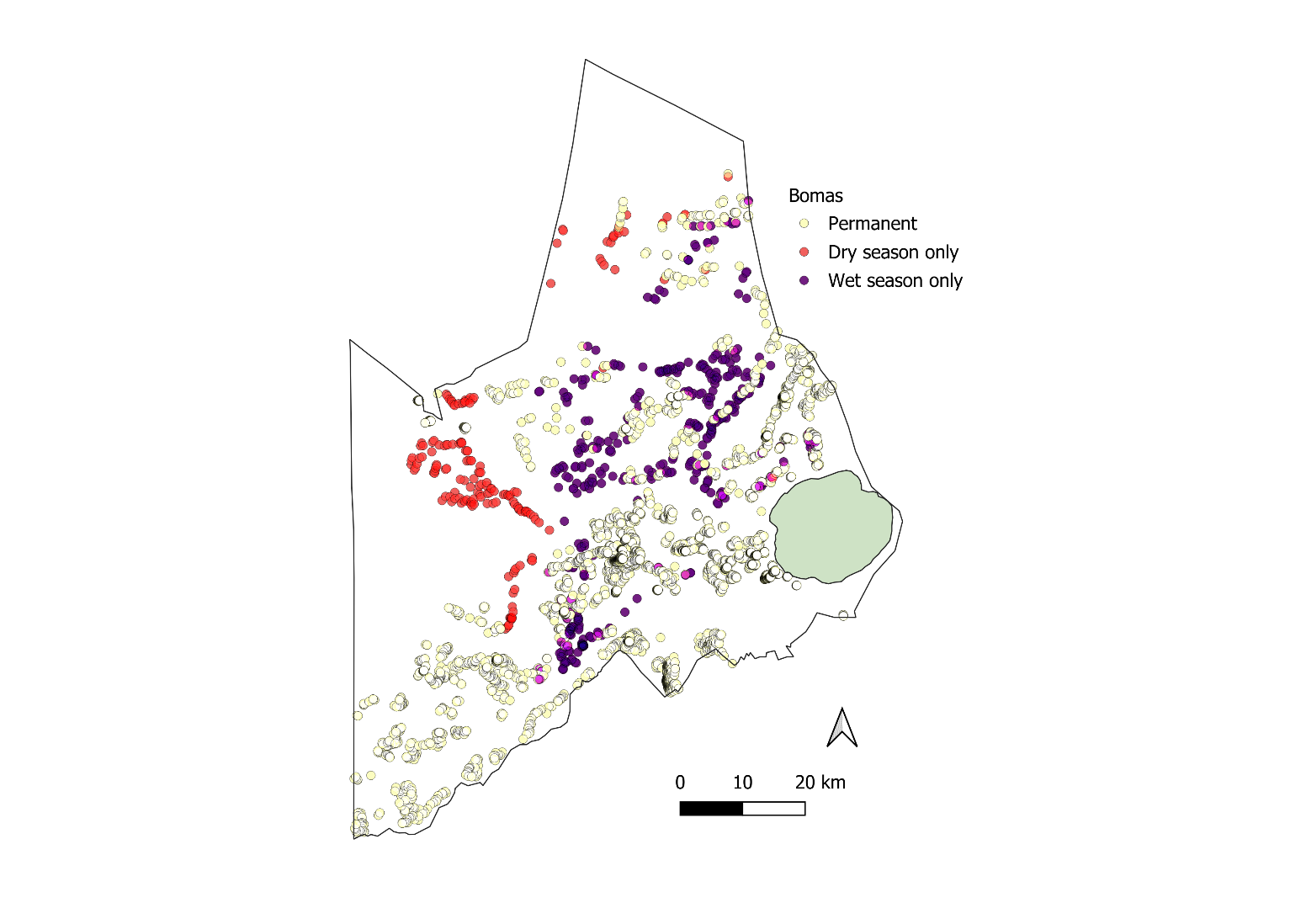

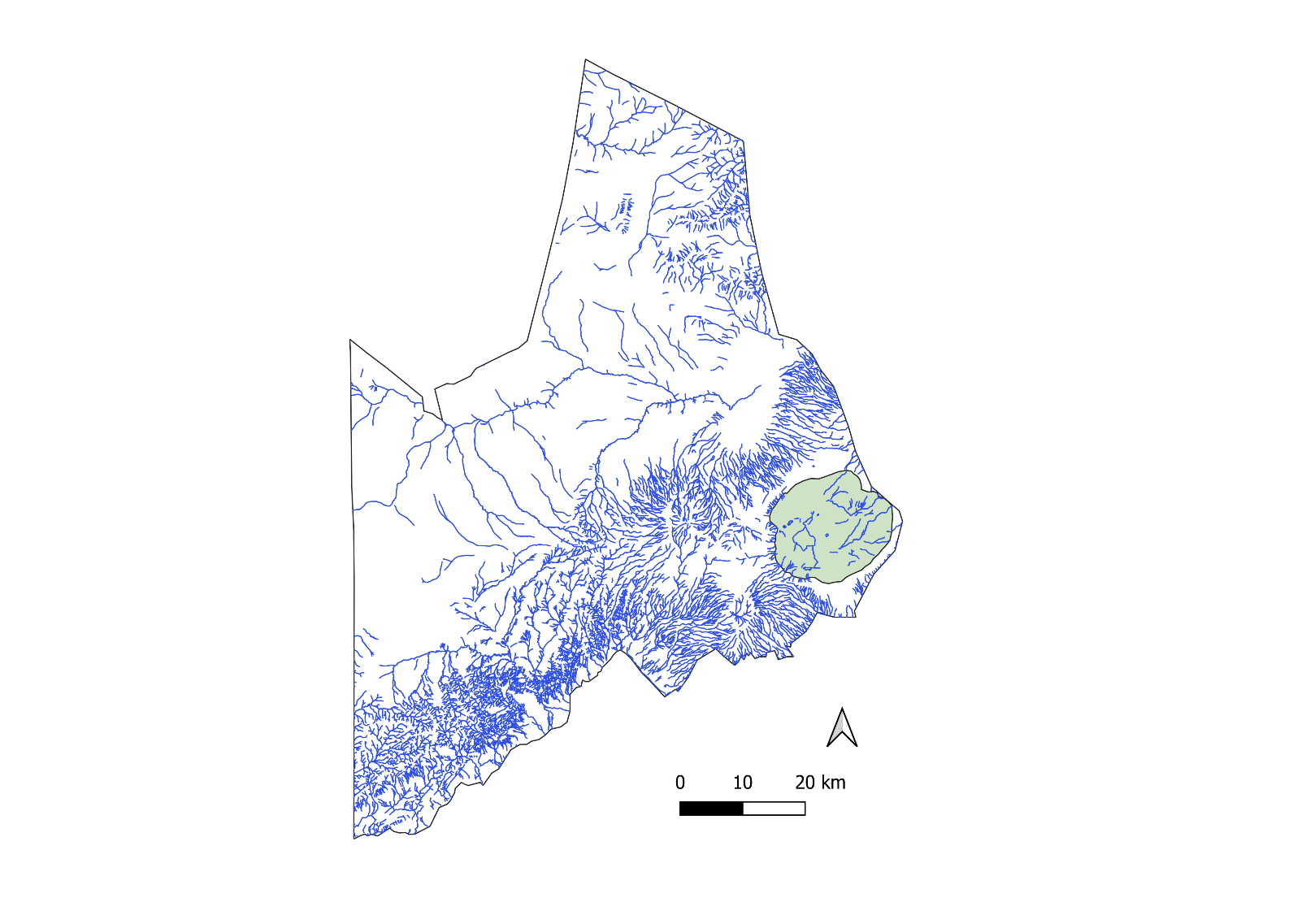


B

A

**S2 Figure.** Study area in the Ngorongoro Conservation Area showing the distribution of rivers (A) and bomas (B). The green polygon is the Ngorongoro Crater.
