# Supplementary material for: Coexistence from a lion’s perspective: Movements and habitat selection by African lions (Panthera leo) across a multi-use landscape
Source: PLoS One. 2024 Oct 3;19(10):e0311178. doi: 10.1371/journal.pone.0311178 (PMC11449311; doi:10.1371/journal.pone.0311178)
Supplement: S4 Fig — The observed distribution of each covariate at the presence points in the test dataset is given by the solid black lines, with associated 95% simulation envelope in gray. The available habitat across the landscape relative to each covariate is given in the dashed red line. Models were well-calibrated, with the observed distributions (solid black lines) falling predominantly within the gray simulation envelopes. Dens_human represents intensity of human activity while Dist_human represents distance to human activity. (DOCX) [file pone.0311178.s008.docx]

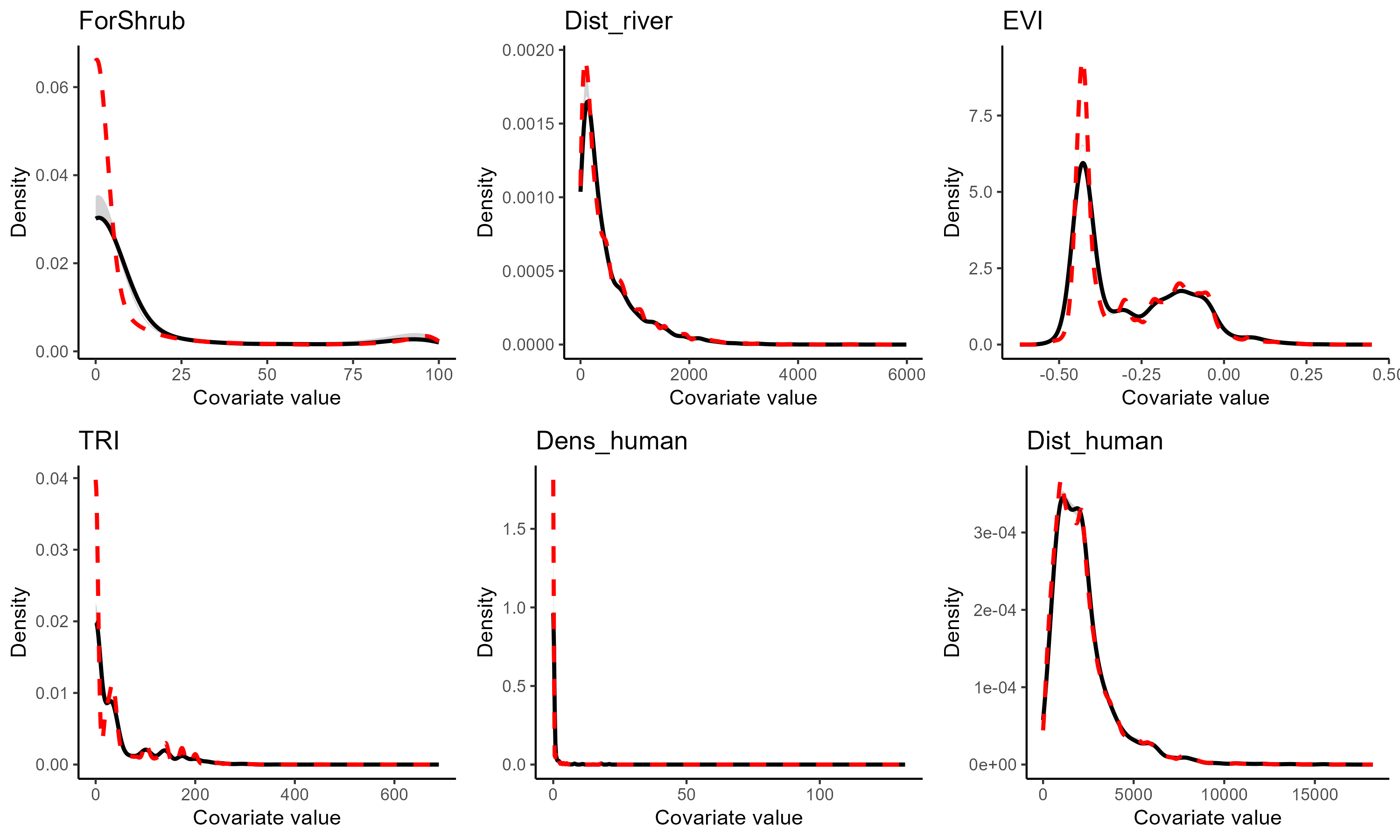


**S4 Figure.** UHC plots for the top step-selection model for resident male lions in Ngorongoro Conservation Area, Tanzania. The observed distribution of each covariate at the presence points in the test dataset is given by the solid black lines, with associated 95% simulation envelope in gray. The available habitat across the landscape relative to each covariate is given in the dashed red line. Models were well-calibrated, with the observed distributions (solid black lines) falling predominantly within the gray simulation envelopes. Dens_human represents intensity of human activity while Dist_human represents distance to human activity.
