# Supplementary material for: Coexistence from a lion’s perspective: Movements and habitat selection by African lions (Panthera leo) across a multi-use landscape
Source: PLoS One. 2024 Oct 3;19(10):e0311178. doi: 10.1371/journal.pone.0311178 (PMC11449311; doi:10.1371/journal.pone.0311178)
Supplement: S6 Fig — We have standardized the y-axis to the level of females for ease of interpretation, please note that means and 95% confidence intervals for resident and nomadic males extend beyond this level. (DOCX) [file pone.0311178.s010.docx]

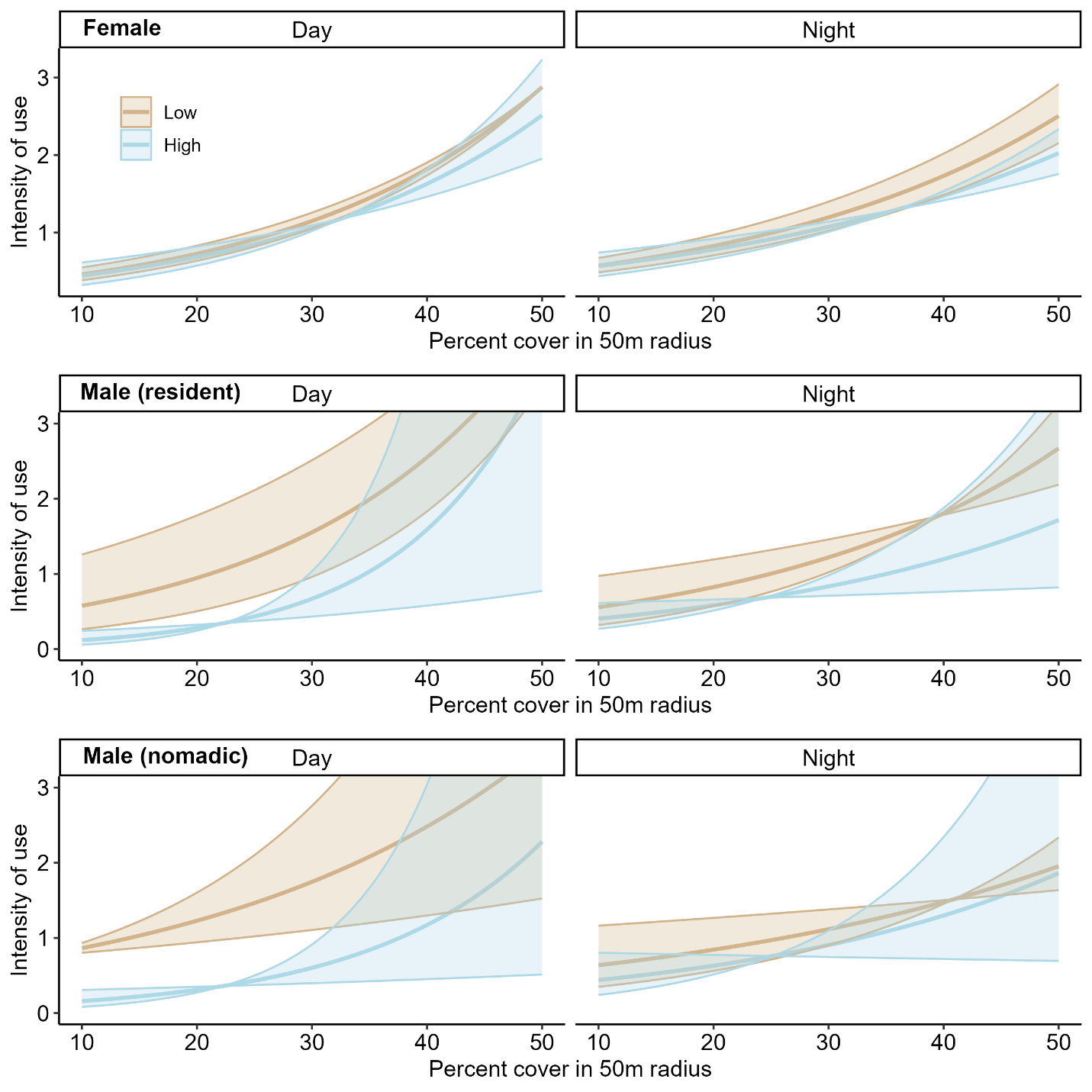


**S6 Figure.** Selection for degree of forest/shrub cover when in areas of either a high (150/km^2^) or low (20/km^2^) density of humans (colors) based on the time of day (facets) for female, resident male and nomadic male lions. We have standardized the y-axis to the level of females for ease of interpretation, please note that means and 95% confidence intervals for resident and nomadic males extend beyond this level.
