# Supplementary material for: Coexistence from a lion’s perspective: Movements and habitat selection by African lions (Panthera leo) across a multi-use landscape
Source: PLoS One. 2024 Oct 3;19(10):e0311178. doi: 10.1371/journal.pone.0311178 (PMC11449311; doi:10.1371/journal.pone.0311178)
Supplement: S8 Fig — Dens_human represents intensity of human activity while Dist_human represents distance to human activity. Relative selection strength was highest for habitat variable Cover (the percent of forest/shrub cover in a 50m radius) for females and resident males. For nomadic males, the variable with the highest relative selection strength was the distance to human activity. (DOCX) [file pone.0311178.s012.docx]

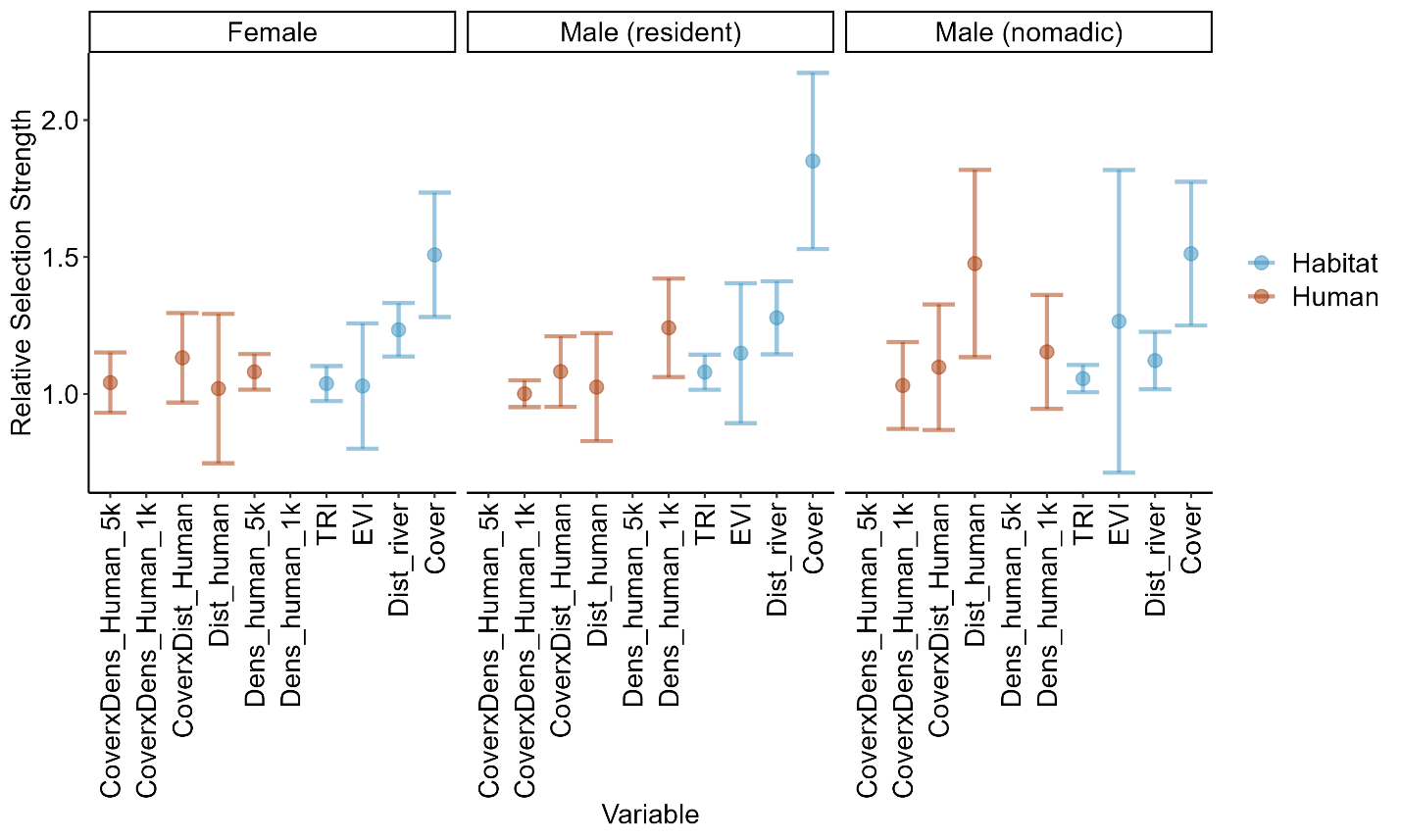


**S8 Figure.** Relative selection strength for variables based on a local scale habitat selection model (SSF) for three classes of lion (females, nomadic males and resident males) in the Ngorongoro Conservation Area, Tanzania. Dens_human represents intensity of human activity while Dist_human represents distance to human activity. Relative selection strength was highest for habitat variable Cover (the percent of forest/shrub cover in a 50m radius) for females and resident males. For nomadic males, the variable with the highest relative selection strength was the distance to human activity.
