# Supplementary material for: Coexistence from a lion’s perspective: Movements and habitat selection by African lions (Panthera leo) across a multi-use landscape
Source: PLoS One. 2024 Oct 3;19(10):e0311178. doi: 10.1371/journal.pone.0311178 (PMC11449311; doi:10.1371/journal.pone.0311178)
Supplement: S10 Fig — (DOCX) [file pone.0311178.s014.docx]

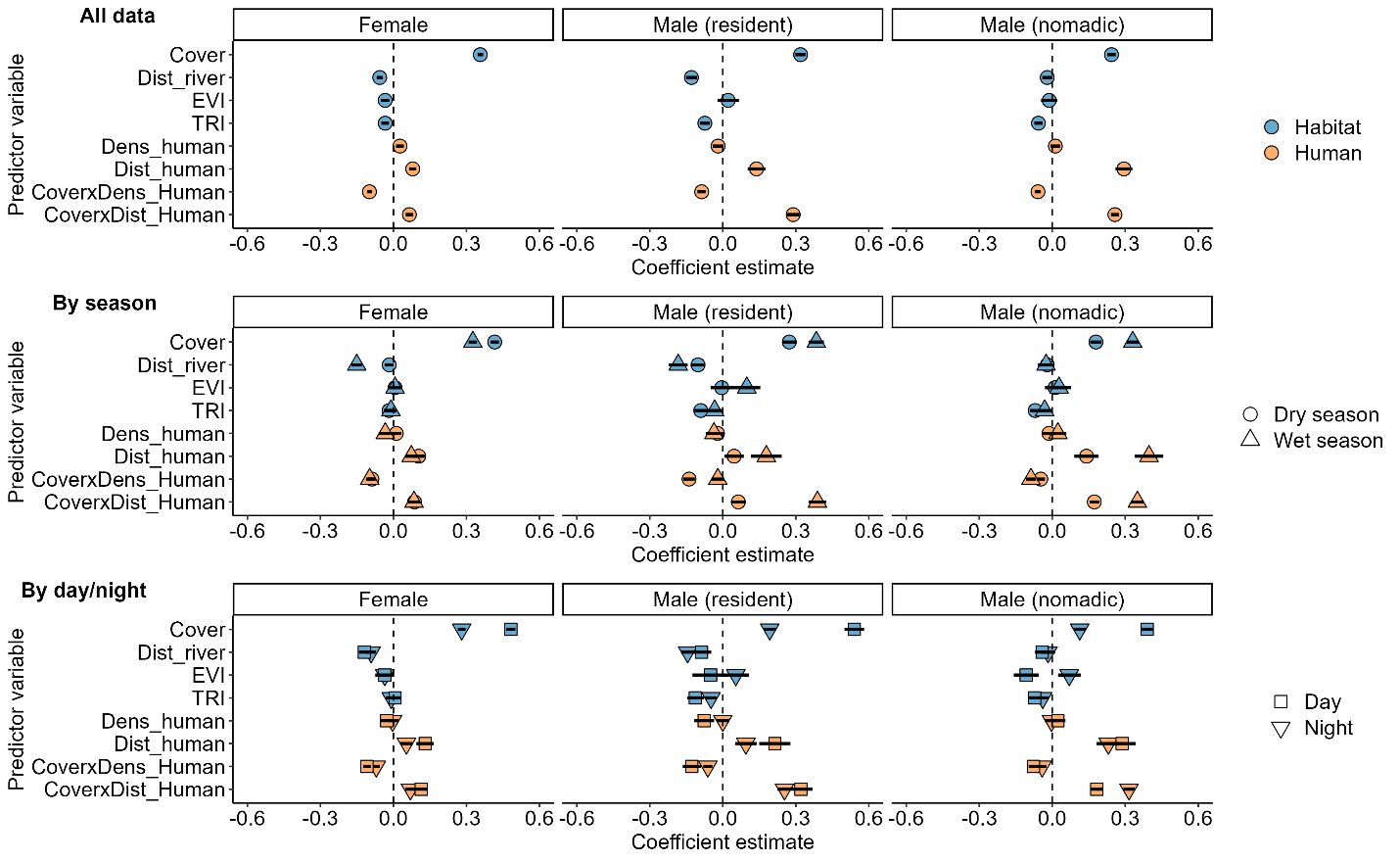


**S10 Figure.** SSF without random slopes, showing much lower variation compared to when individual variation in relationships with predictors is accounted for (Fig. 3).
