# Supplementary material for: Coexistence from a lion’s perspective: Movements and habitat selection by African lions (Panthera leo) across a multi-use landscape
Source: PLoS One. 2024 Oct 3;19(10):e0311178. doi: 10.1371/journal.pone.0311178 (PMC11449311; doi:10.1371/journal.pone.0311178)
Supplement: Attachment — Submitted filename: PONE-D-24-04716_reviewed.pdf [file pone.0311178.s015.pdf]

PLOS ONE
Date: 3rd February 2024

Title: Coexistence from a lion’s perspective: movements and habitat selection by African lions (Panthera leo) across a multi-use landscape

--Manuscript Draft--

**Manuscript Number:**	PONE-D-24-04716
**Article Type:**	Research Article
**Full Title:**	Date: 3rd February 2024<br><br>Title: Coexistence from a lion’s perspective: movements and habitat selection by African lions (Panthera leo) across a multi-use landscape
**Short Title:**	Habitat selection and movements by African lions (Panthera leo) across a multi-use landscape
**Corresponding Author:**	Ingela Jansson, MSc<br>Swedish University of Agricultural Sciences: Sveriges lantbruksuniversitet<br>Umeå, SWEDEN
**Keywords:**	carnivore conservation; connectivity; dispersal; human-wildlife conflict; Ngorongoro Conservation Area; resource-selection function; step-selection function
**Abstract:**	Diminishing wild space and population fragmentation are key drivers of large carnivore declines worldwide. Persistence of large carnivores in fragmented landscapes often depends on the ability of individuals to move between separated subpopulations for genetic exchange and recovery from stochastic events. Where separated by anthropogenic landscapes, subpopulations’ connectivity hinges on the area's socio-ecological conditions for coexistence and dispersing individuals' behavioral choices. Using GPS-collars and resource- and step-selection functions, we explored African lion (Panthera leo) habitat selection and movement patterns to better understand lions’ behavioral adaptations in a landscape shared with pastoralists. We conducted our study in the Ngorongoro Conservation Area, Tanzania, a multiuse rangeland, that connects the small, high density lion subpopulation of the Ngorongoro Crater with the extensive Serengeti lion population. Landscape use by pastoralists and their livestock varies seasonally, driven by the availability of pasture, water, and disease avoidance. The most important factor for lion habitat selection was the amount of vegetation cover, followed by the distance to human settlements and the interaction between those two variables, with selectivity strengths varying with season and time of day. All lions were more willing to approach human settlements at night and during the dry season, selecting strongly for cover when moving closer to humans during the day. Resident females most consistently used areas close to humans, but also relied more consistently on cover than males. Connectivity of lion subpopulations, facilitated by nomadic males, does not appear to be blocked by sparse pastoralist settlements and nomadic males avoided humans on the landscape more strongly than did resident lions. These results are consistent with lions balancing risk from humans with exploitation of livestock by altering their behaviors to reduce potential conflict. Our study lends some optimism for the adaptive capacity of lions to promote coexistence with humans in shared landscapes.
**Order of Authors:**	Ingela Jansson, MSc
	Arielle W. Parsons, PhD
	Navinder J. Singh, PhD
	Lisa Faust, PhD
	Bernard M Kissui, PhD
	Ernest E. Mjingo, PhD
	Camilla Sandström, Docent

Powered by Editorial Manager® and ProduXion Manager® from Aries Systems Corporation

	Göran Spong, PhD
**Additional Information:**	
**Question**	**Response**
**Financial Disclosure**<br><br>Enter a financial disclosure statement that describes the sources of funding for the work included in this submission. Review the submission guidelines for detailed requirements. View published research articles from *PLOS ONE* for specific examples.<br><br>This statement is required for submission and **will appear in the published article** if the submission is accepted. Please make sure it is accurate.<br><br>**Funded studies**<br>Enter a statement with the following details:<br>• Initials of the authors who received each award<br>• Grant numbers awarded to each author<br>• The full name of each funder<br>• URL of each funder website<br>• Did the sponsors or funders play any role in the study design, data collection and analysis, decision to publish, or preparation of the manuscript?<br><br>Did you receive funding for this work?	Yes
Please add funding details.<br>as follow-up to "**Financial Disclosure**<br><br>Enter a financial disclosure statement that describes the sources of funding for the work included in this submission. Review the submission guidelines for detailed requirements. View published research articles from *PLOS ONE* for specific examples.<br><br>This statement is required for submission and **will appear in the published article** if the submission is accepted. Please make sure it is accurate.	The project was supported by grant from the Swedish Research Council, awarded to GS [grant No. 2014-03382]. IJ also was awarded several grants over the years 2012-2023 for operations and equipment linked to this research: yearly grants from NABU International (https://en.nabu.de/about/index.html); National Geographic Society (https://www.nationalgeographic.org/society/) through the Waitt Foundation Grant awarded to C. Packer, and managed by IJ [grant No. W235-12]; National Geographic Society through the Big Cat Initiative [grant No. B15-15, and No. B4-17]; Panthera (years 2012, 2017-2019, https://panthera.org/); Wildaid (years 2017-2019, https://wildaid.org/); Lion Recovery Fund (https://lionrecoveryfund.org/) [grant No. TZ-KL-01, TZ-KL-02-LRF]; SOS – IUCN Save Our Species (https://iucnsos.org/) [grant No. 2021A-6]. The funders had no role in study design, data collection and analysis, decision to publish, or preparation of the manuscript.

Powered by Editorial Manager® and ProduXion Manager® from Aries Systems Corporation

**Funded studies**<br>Enter a statement with the following details:<br>• Initials of the authors who received each award<br>• Grant numbers awarded to each author<br>• The full name of each funder<br>• URL of each funder website<br>• Did the sponsors or funders play any role in the study design, data collection and analysis, decision to publish, or preparation of the manuscript?<br><br>Did you receive funding for this work?"	
Please select the country of your main research funder (please select carefully as in some cases this is used in fee calculation).<br>as follow-up to "**Financial Disclosure**<br><br>Enter a financial disclosure statement that describes the sources of funding for the work included in this submission. Review the submission guidelines for detailed requirements. View published research articles from *PLOS ONE* for specific examples.<br><br>This statement is required for submission and **will appear in the published article** if the submission is accepted. Please make sure it is accurate.<br><br>**Funded studies**<br>Enter a statement with the following details:<br>• Initials of the authors who received each award<br>• Grant numbers awarded to each author<br>• The full name of each funder<br>• URL of each funder website<br>• Did the sponsors or funders play any role in the study design, data collection and analysis, decision to publish, or preparation of the manuscript?<br><br>Did you receive funding for this work?"	SWEDEN - SE
**Competing Interests**	The authors have declared that no competing interests exist.

Use the instructions below to enter a competing interest statement for this submission. On behalf of all authors, disclose any competing interests that could be perceived to bias this work—acknowledging all financial support and any other relevant financial or non-financial competing interests.<br><br>This statement is required for submission and **will appear in the published article** if the submission is accepted. Please make sure it is accurate and that any funding sources listed in your Funding Information later in the submission form are also declared in your Financial Disclosure statement.<br><br>View published research articles from *PLOS ONE* for specific examples.<br><br>**NO authors have competing interests**<br><br>Enter: *The authors have declared that no competing interests exist.*<br><br>**Authors with competing interests**<br><br>Enter competing interest details beginning with this statement:<br><br>*I have read the journal's policy and the authors of this manuscript have the following competing interests: [insert competing interests here]*<br><br>* typeset	
**Ethics Statement**<br><br>Enter an ethics statement for this submission. This statement is required if the study involved:<br><br>• Human participants<br>• Human specimens or tissue<br>• Vertebrate animals or cephalopods<br>• Vertebrate embryos or tissues	Our project included immobilization and capturing of African lion to deploy or exchange GPS-collars, here in Ngorongoro Conservation Area (NCA), Tanzania. We did all collar removals remotely using the collars drop-off function). For this activity we fully adhered to the wildlife handling regulations and policies here in Tanzania. Regulations cites as [Tanzania Wildlife Research Institute (Conduct of Wildlife Research) Regulations, 2020. PART V, IMMOBILISATION, COLLARING AND PHYSICAL HANDLING OF ANIMALS, section. 30-31], accessed via (https://www.tawiri.or.tz/tanzania-wildlife-research-institute-conduct-of-wildlife-research-regulations/)<br><br>Accoring to wildlife research regulations in Tanzania, a research permit is preceeded

Powered by Editorial Manager® and ProduXion Manager® from Aries Systems Corporation

• Field research<br><br>Write "N/A" if the submission does not require an ethics statement.<br><br>General guidance is provided below. Consult the submission guidelines for detailed instructions. **Make sure that all information entered here is included in the Methods section of the manuscript.**	by an accepted research proposal to Tanzania Wildlife Research Institute (TAWIRI), for research permit issued by Tanzania Comission for Science and Technology (COSTECH). IJ has been granted yearly research permits from COSTECH for this research project titled "Balancing Pastoralist Livelihoods and wildlife management in Ngorongoro" since 2011. Here including the permit No's. for the last two permits: 2022-771-NA-2019-068 (Date 13/9/2022) and 2023-793-ER-2019-068 (Date 11/9/2023). This research proposal and permit also include any permission for animal handling, in our case for deploying GPS salellite collars on lions.<br>After permission from TAWIRI and COSTECH, and according to the Regulations, we then received permission from the local authorities in the protected area we work in, in our case the NCA Authority. We have been granted two permissions:<br>Letter No. NCAA/D/240/VOL.XXI/78, Date 30/10/2012, permission to collar up to 6 lions simultaneously.<br>Letter No. BD/158/711/01'E'/54, Date 14/12/2021, permission renewed, with approval to collar up to 8 lions simultaneously<br><br>Any animal handling in Tanzania (capture, immobilization, handling of hte animal while immobilized), is conduted by a Tanzanian registered veterinarian, provided either by TAWIRI or the local authority, in our case, the NCA Authority. This veterinarian has the outmost responsipility for this animals wellfare. The role of us experienced field researchers is to locate the lion, fully attend the immobilisation and handling event (incl deploy collars, take measurements), and stay with the animal until we are confident it is alert, active and deemed well able to defend itself. Further, we monitor positions and movements of our active collars on a daily basis - and apart from research in lions finescale movements, these collars have become a very important tool for preventing human-lion conflict and thus improves lions' safety.<br><br>The collars weigh between 1-1.2 kg - which 1% or less of an adult lions bodyweight, and deemed not to harm an animal. From our close monitoring, we have never seen any negative effects from any of our collars on the lions (incl in behaviours like hunting, feeding, socilizing, mating, reproducing). We only target fully grown lions for immobilization and collaring.<br><br>The lions were immobilized using a dart-gun from a vehicle at 10–20-meter distance. Lions in NCA community lands (the ones we targeted for collaring) are few and elusive, hence immobilization to deploy collars is challenging and done opportunistically, mainly at night, following observations of lions in the area. To attract lions closer to the vehicle for immobilization, we broadcasted a high-volume recording of feeding hyenas, a bleating buffalo calf, or the roars of a lion. The drugs used in for the immobilization was typically a mixture of zoletil and medetomidin, and for its antidote a mix of yohimbine ana atipamazole (dose adjusted depending on size of lion). Up until 2018 Ketamin was sometimes used.

Powered by Editorial Manager® and ProduXion Manager® from Aries Systems Corporation

**Format for specific study types**<br><br>**Human Subject Research (involving human participants and/or tissue)**<br>• Give the name of the institutional review board or ethics committee that approved the study<br>• Include the approval number and/or a statement indicating approval of this research<br>• Indicate the form of consent obtained (written/oral) or the reason that consent was not obtained (e.g. the data were analyzed anonymously)<br><br>**Animal Research (involving vertebrate animals, embryos or tissues)**<br>• Provide the name of the Institutional Animal Care and Use Committee (IACUC) or other relevant ethics board that reviewed the study protocol, and indicate whether they approved this research or granted a formal waiver of ethical approval<br>• Include an approval number if one was obtained<br>• If the study involved *non-human primates*, add *additional details* about animal welfare and steps taken to ameliorate suffering<br>• If anesthesia, euthanasia, or any kind of animal sacrifice is part of the study, include briefly which substances and/or methods were applied<br><br>**Field Research**<br><br>Include the following details if this study involves the collection of plant, animal, or other materials from a natural setting:<br>• Field permit number<br>• Name of the institution or relevant body that granted permission	
**Data Availability**<br><br>Authors are required to make all data underlying the findings described fully available, without restriction, and from the time of publication. PLOS allows rare exceptions to address legal and ethical concerns. See the PLOS Data Policy and FAQ for detailed information.	Yes - all data are fully available without restriction

A Data Availability Statement describing where the data can be found is required at submission. Your answers to this question constitute the Data Availability Statement and **will be published in the article**, if accepted.<br><br>**Important:** Stating 'data available on request from the author' is not sufficient. If your data are only available upon request, select 'No' for the first question and explain your exceptional situation in the text box.<br><br>Do the authors confirm that all data underlying the findings described in their manuscript are fully available without restriction?	
**Describe where the data may be found in full sentences. If you are copying our sample text, replace any instances of XXX with the appropriate details.**<br><br>• If the data are **held or will be held in a public repository**, include URLs, accession numbers or DOIs. If this information will only be available after acceptance, indicate this by ticking the box below. For example: *All XXX files are available from the XXX database (accession number(s) XXX, XXX.*).<br>• If the data are all contained **within the manuscript and/or Supporting Information files**, enter the following: *All relevant data are within the manuscript and its Supporting Information files.*<br>• If neither of these applies but you are able to provide **details of access elsewhere**, with or without limitations, please do so. For example:<br><br>*Data cannot be shared publicly because of [XXX]. Data are available from the XXX Institutional Data Access / Ethics Committee (contact via XXX) for researchers who meet the criteria for access to confidential data.*<br><br>*The data underlying the results presented in the study are available from (include the name of the third party*	The one large datafile, containing all data for the analyses in this manuscript is available from the Dryad database (accession number https://doi.org/10.5061/dryad.j6q573nnb)<br>For the option "Choose when to publish", I selected for now to "Keep my dataset private while my related article is in peer review". Once/if submitted manuscript is accepted for review, I will make it openly available.

and contact information or URL).<br>• This text is appropriate if the data are owned by a third party and authors do not have permission to share the data.<br><br>* typeset	
Additional data availability information:	

Manuscript
Click here to access/download;Manuscript;Jansson_et_al_lion_habitat_selecti

Coexistence from a lion's perspective: movements and habitat selection by African lions (*Panthera leo*) across a multi-use landscape

Ingela Jansson[1,2*¶], Arielle W. Parsons[3¶], Navinder J. Singh[1], Lisa Faust[3], Bernard M. Kissui[4], Ernest E. Mjingo[5], Camilla Sandström[6], Göran Spong[1]

[1] Molecular Ecology Group, Department of Wildlife, Fish and Environmental Studies, Swedish University of Agricultural Sciences, Umeå, Sweden

[2] KopeLion, Inc., Ngorongoro Conservation Area, Arusha, Tanzania

[3] Alexander Center for Applied Population Biology, Lincoln Park Zoo, Chicago, IL, USA

[4] School for Field Studies, Karatu, Tanzania

[5] Tanzania Wildlife Research Institute (TAWIRI), Arusha, Tanzania

[6] Department of Political Science, Umeå University, Umeå, Sweden

[*]Corresponding author

E-mail: ingela.jansson@slu.se (IJ)

[¶]These authors contributed equally to this work.

Abstract

Diminishing wild space and population fragmentation are key drivers of large carnivore declines worldwide. Persistence of large carnivores in fragmented landscapes often depends on the ability of individuals to move between separated subpopulations for genetic exchange and recovery from stochastic events. Where separated by anthropogenic landscapes, subpopulations’ connectivity hinges on the area's socio-ecological conditions for coexistence and dispersing individuals' behavioral choices. Using GPS-collars and resource- and step-selection functions, we explored African lion (*Panthera leo*) habitat selection and movement patterns to better understand lions’ behavioral adaptations in a landscape shared with pastoralists. We conducted our study in the Ngorongoro Conservation Area, Tanzania, a multiuse rangeland, that connects the small, high density lion subpopulation of the Ngorongoro Crater with the extensive Serengeti lion population. Landscape use by pastoralists and their livestock varies seasonally, driven by the availability of pasture, water, and disease avoidance. The most important factor for lion habitat selection was the amount of vegetation cover, followed by the distance to human settlements and the interaction between those two variables, with selectivity strengths varying with season and time of day. All lions were more willing to approach human settlements at night and during the dry season, selecting strongly for cover when moving closer to humans during the day. Resident females most consistently used areas close to humans, but also relied more consistently on cover than males. Connectivity of lion subpopulations, facilitated by nomadic males, does not appear to be blocked by sparse pastoralist settlements and nomadic males avoided humans on the landscape more strongly than did resident lions. These results are consistent with lions balancing risk from humans with

exploitation of livestock by altering their behaviors to reduce potential conflict. Our study lends some optimism for the adaptive capacity of lions to promote coexistence with humans in shared landscapes.

Introduction

Large carnivores range over vast areas, requiring a mix of habitats to fulfil their basic needs of food, shelter, and reproduction. In a world with diminishing wild spaces, carnivore ranges increasingly overlap with humans, impinging movements [1] and leading to conflicts and persecution [2]. In many cases, anthropogenic encroachment is extreme enough to fragment large carnivore populations into small, increasingly isolated subpopulations, jeopardizing their long-term viability and persistence [2–4]. Such populations are more likely to experience negative effects from stochastic events, genetic drift, and inbreeding, leading to lower levels of genetic variation. Ultimately, these effects increase extinction risk by enhancing vulnerability to environmental change and disease and/or reducing long-term health and fecundity [3].

The long-term persistence of large carnivores in fragmented landscapes often depends on the ability of individuals to disperse between isolated subpopulations which serves to reduce population-level genetic drift, inbreeding depression, and the risk of extinction due to stochastic events [5]. Navigating fragmented landscapes involves using and traversing habitats dominated by humans (i.e., shared landscapes). Shared landscapes may provide carnivores with ample resources (e.g., livestock) and reduced intra-guild competition [6] but also contain new risk-factors from direct conflict with people (e.g., persecution by hunting or poisoning), making them potential sink habitats [7]. Spatiotemporal scarcity of natural prey may also push

carnivores to more risky behaviors, including increased overlap with people [8]. One of the main factors promoting successful connectivity for carnivores is management to enhance coexistence between humans and wildlife [9–13]. Large carnivore studies have frequently demonstrated increased mortality risk of dispersing individuals through fragmented landscapes, especially those occupied by humans [14]. Efforts to make human-free corridors for wildlife movement are becoming less feasible as human populations grow globally and encroach on wild habitats [1]. Thus, finding ways to promote coexistence and facilitate connectivity through movement across human-occupied habitats is becoming increasingly important for the conservation of wildlife populations.

The African lion (*Panthera leo*) is adaptable to a variety of habitats, but soon ceases to persist in landscapes dominated by humans [15,16]. Human land-uses compatible with naturally functioning landscapes, such as pastoralism, offer opportunities for coexistence with wildlife including lions [17,18]. However, lions living among pastoralists are especially vulnerable to conflict through retaliatory killings, due to their habits of primarily preying on high value cattle (*Bos taurus*), attacking during the daytime, and lingering by the kill. Other main livestock predators, the spotted hyena (*Crocuta crocuta*) and leopard (*Panthera pardus*) tend to prey upon lower-value livestock, attack at night, and be more elusive to better escape persecution [19]. In addition, traditional spearing of lions to demonstrate bravery is practiced among some pastoralist cultures, e.g. the Maasai [20,21], contributing to human-lion conflict and the lions' fear of people. However, lions have an established capacity for flexibility, exhibiting behavioral adaptations to avoid conflict with humans (e.g., becoming more nocturnal [9,22]), often resulting in improved survival. Whether these behaviors can improve success in

connecting isolated subpopulations remains unclear, however understanding the behavioral choices of lions in shared landscapes will be critical for making the best decisions for lion conservation as anthropogenic landscapes expand across Africa.

Lions are a group-living species with a complex social structure [23,24]. Lions of different sexes and life stages behave differently, making unique behavioral choices based on landscape familiarity and response to threats [24,25]. While females are philopatric and territorial, males depart from their natal prides at maturity, becoming nomadic until they are able to gain resident status with a pride. A male's resident state in a pride is temporary, typically 2-3 years, after which they are out-competed by other males or leave voluntarily, re-entering a nomadic phase [24]. Nomadic males don't hold territories and are subordinate to resident males, wandering widely in search of females and often relegated to suboptimal habitats to avoid conflicts with other lions [24,26]. Carnivore studies rarely consider these sex and life-stage differences when assessing habitat use and thus might miss important nuances of behavior and movements relevant to human-wildlife conflict.

Here, we present a study of the lion population of the Ngorongoro Conservation Area (NCA), Tanzania. The NCA is an important component of the Greater Serengeti Ecosystem and its only multiuse protected area, where traditional pastoralists and their livestock (cattle, sheep (*Ovis aries*), goats (*Capra hircus*), and donkeys (*Equus asinus*)) share the landscape with wildlife. The NCA's multiuse area connects the small, high density lion subpopulation of the Ngorongoro Crater with the extensive Serengeti National Park (NP). Increasing human activity and human-lion conflicts have been a main driver for lion population decline and disappearance across the NCA, halting dispersal and increasing the isolation of the Ngorongoro Crater

subpopulation [21,27]. Using data from GPS collars, we tested the best predictors of lion habitat use across the NCA at two spatial scales (local/landscape), within two seasons (wet/dry) and diel periods (night/day). To account for and understand differences between sexes and life-stages, we analyzed habitat use separately for both resident lions of each sex, and for nomadic males. Our goal was to understand how lion habitat selection choices are affected by the benefits of ideal habitats and risks of human activity. We hypothesize that the intensity of anthropogenic presence reduces lion use of the shared landscape, especially for nomadic lions, but that lions mitigate risk through behavioral changes. Specifically, we predict that while environmental factors will influence lion landscape-scale habitat selection and local movements, anthropogenic factors will have the largest associated effects. We also predict that the response of lions to humans as lions move through and use the landscape will change at night, between the seasons and with natural habitat availability. Specifically, we predict higher landscape-scale avoidance of people during the wet season, despite lower intensity of use by humans during this time, due to more abundant and widespread natural prey. However, at the local scale (i.e. the immediate surrounding habitat) we expect lower avoidance of people during the wet season, at night and in areas of ample cover for hiding.

Methods

Study area

The Ngorongoro Conservation Area (NCA) in Tanzania (-3°E and 35°N) is an 8,300 km^2 component of the Greater Serengeti Ecosystem. The NCA is unique in being a multiple-use

protected area that allows traditional pastoralists and their livestock to co-exist with wildlife, including lions [28]. People of the Maasai and Datooga (Barabaig) ethnic groups reside here at a population density of roughly 12/km^2, of which the great majority subsist mostly or entirely on livestock. While most of the NCA is accessible for grazing and settlement, some areas are excluded for wildlife use only: the craters (including the Ngorongoro Crater), the eastern highland forest, and a section called Ndutu near the Serengeti NP border (Fig. 1). The landscape is a mix of forested highlands, rugged escarpments, volcanic calderas, *Acacia* and *Commiphora* woodland savannah, and vast open grasslands. Altitudes range between 1,000 - 3,000 masl, with a west to east rainfall gradient between 400 - 700 mm/year. The eastern highlands are foggy and cool during Jun-Aug, with nighttime temperatures often as low as +5°C. In the west, the vast short-grass plains form a seamless border to Serengeti NP – barren for much of the year, these plains provide highly nutritious pasture during the rains [29], a key driver of the great Serengeti ungulate migration [30]. NCA's varied topography, climate, vegetation, and seasons create a rich and heterogeneous landscape for abundant wildlife and provide valuable pastures for livestock. The area of this study encompassed approximately 4,800 km^2 of the central expanse of the NCA, from and including the Ngorongoro Crater westward to Ndutu and the border of Serengeti NP (Fig. 1).

Fig 1: Map of Ngorongoro Conservation Area and the Greater Serengeti Ecosystem, northern Tanzania.

Ngorongoro Conservation Area (NCA, black outline) is a large component of the Greater Serengeti Ecosystem (blue outline), Tanzania. While most of the NCA is multi-use, allowing

pastoralists to coexist with wildlife, there are several areas reserved for wildlife only (in orange). The area of this study (dotted black line) encompassed around 4,800 km^2 of the central expanse of the NCA, from and including the Ngorongoro Crater westward to Ndutu and the border of Serengeti National Park to the north.

The NCA exhibits wet and dry seasonality, with a typical pattern of “short rains” Nov-Dec and “long rains” Mar-Apr [29]. The rains’ time-lag effects on the pasture, a key resource for both wildlife and livestock, provides good wet-season grazing between Dec-May, and limited dry-season grazing between Jun-Nov. During the dry season, water is scarce, with most rivers being seasonal and many water-bodies undrinkable due to very high soda/salt levels. Consequently, wild herbivore abundance is low during the dry season and pastoralist movements intensify in search of pasture yet remain restricted to areas close to available water. Aside from the herders moving between temporary homesteads, NCA’s pastoralist families have become increasingly sedentary [31] and the majority reside in permanent homesteads. When the rains arrive, vast herds of migratory ungulates, including ~1.5 million wildebeest (*Connochaetes taurinus*) [32,33], return to the shortgrass plains which covers ~40% of the NCA on its border to Serengeti. Between Jan – Apr, during the wildebeest’s synchronized calving [34], pastoralists remove their cattle herds from the shortgrass plains to avoid infection from Malignant Catarrhal Fever, transmitted by the wildebeest’s placenta to new-born calves [35,36]. However, this virus does not affect sheep and goats, and some spread-out pastoralist settlements and their small stock herds remain. This natural phenomenon creates a period of greatly reduced overlap between pastoralist and their livestock and wildlife on the short-grass

plains in the wet season, which then hosts a super-abundance of wildlife. Thus, lions residing on NCA's shortgrass plains experience a wet season teeming with natural conflict-free prey, and a dry season abundant with risky prey, namely livestock.

Lion movements

To capture lion movements and use of habitat, we deployed satellite GPS collars (models GPS Plus or Vertex Lite by Vectronic Aerospace (www.vectronic-aerospace.com) on lions of both sexes. Between Oct. 2012- Mar. 2023, we had 22 different individuals collared: eight females (190 months total) and 14 males (252 months total). On average, individual lions were collared 19.9 months (range: 1.7 to 56.4 months) with nine lions being collared for two years or more (S1 Table). The collars had Iridium satellite data transmission (for regular data transfer), VHF-beacon (for real-time manual tracking), remote drop-off function, and were scheduled to take positions every 1-2 hours continuously, day or night. The collar batteries lasted for 2-3.5 years, after which the collar was either removed (using remote drop-off) or replaced. We targeted lions for collaring from adult individuals of either sex from outside the Ngorongoro Crater, in NCA's multiuse area, from different groups and areas, based on our knowledge of the usual range of their pride or group. We prioritized lions in areas with heightened risk of conflict. Apart from studying the lions' behavior through fine-scale movements, the purpose of the collars was to provide early-warning to livestock herders in the area.

Lions in NCA community lands are few and elusive, hence immobilization to deploy collars is challenging and done opportunistically, mainly at night, following observations of lions in the area. To attract lions closer to the vehicle for immobilization, we broadcasted a high-

volume recording of feeding hyenas, a bleating buffalo calf, or the roars of a lion. The lions were immobilized and collared under permission from the Tanzania Wildlife Research Institute and the Ngorongoro Conservation Area Authority, whose veterinarians immobilized and supervised the collaring of all animals. Lions were immobilized using a dart-gun from a vehicle at 10–20-meter distance, and collars were fitted by an experienced field researcher. All lions were monitored until alert and deemed safe and well recovered after waking up from the immobilization.

Human activity proxy

To capture human activity across the landscape, our field team, recruited from the community residents based on their high local area knowledge, recorded GPS locations of bomas and water-points (i.e., rivers, springs, wells, and dams with drinkable water) within our study area between 2013-2016. Bomas are a pastoralist family homestead, with a large corral for livestock in the center surrounded by huts and occasionally small houses. Bomas are the center of livestock herding activity, where livestock depart every day for pasture and water, typically ranging up to 10 km per day from bomas. Boma locations included permanent and seasonal bomas (used only in the dry or wet season) with exact locations varying over time even as the general settlement areas have remained the same. Since the area used by nomadic male lions sometimes exceeded our main study area, we expanded the mapping of water-points and bomas to cover long-distance tracks by lions using a combination of remote mapping and ground surveys. For remote mapping, team members most familiar with the area viewed Google Earth satellite maps, assisted by artificially generated building locations [37], to

geolocate bomas and assign them as permanent or seasonal. Using the same procedure, we mapped all buildings within the study-area. Some (33%) of the geolocated bomas were mapped in the field, and had an average 4.6 (StDev 3.44, Median 4, range 0-30) huts per boma. This included both large and small, permanent, and seasonal bomas, and provides a fair representation of boma structures across the NCA. Compared to bomas, buildings are rare in the NCA and clustered within small areas of village centers, schools, hospitals, ranger posts, tourism lodges, and permanent camps. From a lion perspective, human activities from buildings are likely less relevant than bomas due to their lack of livestock activity, but nevertheless represent a concentrated threat from humans. The data on locations of bomas and buildings were combined, resulting in the spatial layer hereafter referred to as “bomas” that we used as a proxy for human activity.

Landscape variables

We modelled lion habitat selection using landscape variables (Table 1) representing habitat characteristics and conflict risk (i.e., humans on the landscape). Habitat covariates included the percent of shrub/forest habitat within a 50m radius (hereafter termed cover) to capture lion preference for sheltering and/or stalking under cover [38,39]; a terrain ruggedness index (hereafter “TRI”) [40] to capture lions’ use of rugged, less accessible areas for shelter and/or stalking; an enhanced vegetation index (hereafter “EVI”; MODIS) to capture greenness as an indication of herbivore (lion prey) abundance; and the distance to the nearest riverbed (S1 Figure) which lions commonly use for stalking prey or resting in thick riverine vegetation [38,41]. Although EVI values for the region are available monthly, to reduce computation time

and model complexity, we summarized EVI for wet (Dec-May) and dry (Jun-Nov) seasons, respectively, by averaging over each year from 2012-2022. We used the month of April to represent the wet season and October to represent the dry season, typically the peak months of greenness for each season [29]. Human-related covariates included the distance to the nearest boma (hereafter “human activity; S1 Figure), the distance to the nearest water point, and the density of bomas within a defined radius (3km, 5km; see below) (hereafter, “intensity of human activity”). Since many bomas and water points shift seasonally, we created separate covariates for distance to human activity, intensity of human activity, and distance to water points to model wet and dry seasons separately.

Table 1: Covariates used for modelling habitat selection of lions in the Ngorongoro Conservation Area, Tanzania

Variable	Source	Resolution	Description
Cover	[37]	50m	The percent of a 50m radius grid cell that contains $4m^2$ pixels classified as “Forest” or “Shrub”, i.e. vegetation cover.
Dens_human	Field mapping, Google Earth, [37]	1km, 3km and 5km.	Intensity of human activity (density of bomas) in 1km, 3km and 5km radius. Since pastoralists move bomas in the wet season, dry- and wet-season layers for each radius were produced.
Dist_human	Field mapping, Google Earth, [37]	300m	Distance (m) to nearest human activity (boma). Since pastoralists move bomas out of certain areas in the wet season, dry- and wet-season layers were produced.
Dist_water	Field mapping	300m	Distance (m) to nearest known water sources. Some water sources are only available in the wet season (we included only sources with water available > 2 months into the dry season), thus dry and wet season layers were produced.
Dist_river	[42] https://serengetidata.weebly.com/ and [43],	300m	Distance (m) to the nearest river course. Rivers are dry most of the year but used by lions as a landscape feature for resting, ambush hunting, denning. A single layer was produced and used in both dry and wet season modelling.

	https://www.hydrosheds.org/		
EVI	MODIS monthly vegetation Indices (MOD13A3) Version 6	1km	Enhanced Vegetation Index (EVI) represents vegetation cover while minimizing canopy background variation. Since EVI depends on precipitation, separate dry- and wet-layers were used.
TRI	Produced from USGS EROS digital elevation model using the terrain ruggedness index of [40]	30-arc seconds	Terrain ruggedness index. Terrain ruggedness varies over the study area and more rugged terrain might provide more shelter for elusive lions. This index ranks the relative ruggedness of the landscape with higher numbers being more rugged.

Movement analysis

Habitat selection patterns are often scale-dependent [44], therefore we analyzed how lions select habitats at the landscape scale and how they then navigate those chosen habitats at the finer local scale. We assessed landscape-scale selection using resource selection functions (RSF), and fine-scale selection using step-selection functions (SSF). For each scale, we ran a separate set of models for three different lion categories: resident males (i.e., those settled with a pride and not making long-distance movements), nomadic males (i.e., males not associated with a pride, making frequent long-range movements) and females. Over the course of monitoring, males could be classified as nomadic or resident at different times, with 10 of the 15 males defined as nomadic during at least one month (S1 Table). Our definitions of the social status of a male were based on our long-term detailed lion monitoring with direct observations and individual recognition [23]. A male was defined as nomadic when he departed from his natal pride (usually by 2-3 years of age), and/or when he ceased to be the resident

male of a pride. A male was defined as a resident when he was seen consecutive times with a pride and actively engaging with the females, a status that typically lasts for 2-3 years [24].

We began by testing the best radius over which to calculate human density (i.e., boma density). Bomas tend to be distributed widely across the landscape (mean distance between nearest neighbors = 3.23km), thus we selected an appropriate radius size to represent this spread (3km). However, we expected different lion categories to respond differently to bomas, based on differences in movement patterns and behavior [45]. Thus, we tested larger and smaller radii for each lion category (1km, 3km and 5km), running a single SSF for each radius for each lion category, that we ranked by Akaike Information Criterion (AIC) to select the most supported radius for subsequent modelling steps. We assessed average movement distances for each category of lion using mean squared displacement (MSD) [46,47]. We modelled the difference in MSD between wet and dry seasons using a generalized linear model with log link to assess the season in which each category of lion moves most. We modelled habitat selection (RSF and SSF) separately for wet and dry seasons and nocturnal and diurnal movements [22], using the R package suncalc [48]. By modelling wet/dry season and daytime/nighttime movements separately, we allowed for different relationships between habitat selection and each covariate between seasons and times of day.

Landscape scale habitat selection

At the landscape scale, we restricted our RSF analysis to the study area over which we had complete datasets for boma and water-points, removing the few exploratory lion tracks that occurred outside of that area. We used package amt [49] in program R [50] to generate random

available locations (n=100 locations for each lion location) within the study area to compare to the locations actually used by each animal. To avoid pseudo-replication and the influence of lengthy resting locations, we resampled the position of each animal at 12-hour intervals. We included random intercepts for each individual animal to account for individual behavioral variation. We used the R package glmmTMB [51] to run our models, fixing the variance of the random intercept for strata to 10^3 [52].

Local scale habitat selection

We used SSFs to quantify lions' habitat selection at the local scale by comparing habitat covariates at locations that the lions had visited with habitat covariates at a random set of available locations (i.e. the habitats available along animal movement tracks) [53]. The available locations for SSF modelling are conditioned on the last step taken by the animal, meaning they are relative to the current location, resulting in a stratified dataset [52]. Again we used the amt package [49] to estimate each animal's movement parameters for step length and turn angle, then sampled from these parameter distributions to generate random available locations (n=50 locations) around each used location. We did not restrict this analysis to the study area, allowing inclusion of long-distance tracks around which we had targeted additional collection of covariate data. We extracted covariate vectors for each location and used the Poisson formulation of conditional logistic regression to estimate associated slope coefficients [54]. We included random intercepts for each strata (i.e., set of used and available steps) [52] and used R package glmmTMB to run our models, fixing the variance of the random intercept for strata to 10^3 [52].

For each lion category (female, resident male, nomadic male), we tested six sub-models. First, we tested a model including all predictors. Next, we tested the value of adding an interaction term to this fully-parameterized model representing the selection for cover relative to human activity (i.e., the distance to and intensity of human activity, respectively). Next, we tested a model including only habitat-related predictors against a model including only human activity predictors. Initial tests with fully-parameterized models indicated the percent of a 50m radius area that provided cover had a strong influence on step-selection for each lion category. We therefore tested two additional habitat models, one where cover was the sole predictor and another where all habitat predictors except for cover were included. Following model-selection, we ran the top model for each lion category separately for each season (wet and dry) and by time of day (day, night) to determine if relationships with habitat and human covariates differed.

We assessed the fit of our top model using used-habitat calibration plots (UHC) via the R package uhcplots [55]. First, we split data for each lion category into random training and testing halves and fit the respective top model using the training dataset. From the fitted model, we randomly sampled 1000 times from the distribution of each slope coefficient, selecting a new slope each time for each covariate in the model. We then used these simulated slope coefficients to generate predicted values. We generated density plots to compare observed and predicted covariate values. We then visually assessed how well observed and predicted covariate distributions overlapped as a representation of model fit [55].

RSS and resistance mapping

Since connectivity between sub-populations is expected to result from male lions which tend to make long-distance movements at initial dispersal, and sometimes throughout their lives [23], we based our resistance mapping solely on nomadic males. We used relative selection strength (RSS) based on our SSF model to calculate habitat resistance for nomadic males across the study area [56]. RSS is the ratio of the probability of a used step being selected to the probability of an available step being selected and is given by the exponential of the estimated slopes in the step-selection model [56]. Habitat resistance is represented by 1-RSS, thus we first calculated RSS for our top model. Following the method of [56,57], we generated a dataset of 200 equally spaced values within each covariate's scaled range. We generated separate ranges for covariates that varied between wet and dry seasons and calculated resistance separately for each season. We then estimated an RSS function (i.e., relative selection curve) for each covariate in each season, given by:

$$RSS_i = \Delta h_i(\beta_i)$$

where Δh_i is the difference between the mock value of covariate *i* at location x_1 and the average attribute value across the landscape (0 for scaled continuous covariates). β_i is the slope coefficient estimate of covariate *i* from our step-selection model for a given season.

The resulting season-specific RSS functions for each covariate were used to predict the resistance of each cell in the study area based on cell-specific covariate values for that season. First, we calculated the RSS value for each cell separately for each covariate, then we subtracted the RSS value from 1 (i.e., resistance) for each covariate in each cell of the study

area. Finally, we summed the resistance values across all covariates for each cell to get an index of total cell resistance in that season. Thus, the resistance value *R* at each cell *i* in a season is given by:

$$R_i = \sum_{n=i}^{\infty} \exp(1 - (\alpha_0 + \alpha_x X_i))$$

where α_0 and α_x are the intercept and slope of the RSS function for covariate *x,* respectively and X_i is the value of covariate *x* at cell *i*.

Results

We found that two variables, distance to the nearest water point and distance to human activity, were highly correlated (0.82), thus we selected only one variable, distance to human activity, for use in our models. When we tested different radii (1km, 3km and 5km) for the calculation of intensity of human activity (i.e. boma density), we found no clear support for any radius over the others for nomadic males (all within 2 AIC points; S3 Table) [58]. For resident males, AIC model selection supported using a 3km radius over other options, while for females the 5km radius was most supported (S3 Table). In lieu of concrete support for any radius for nomadic males, and to be consistent in our modelling of all males, we used a 3km radius for both male categories. On average, males (msd: nomadic males 161.83, SE=37.29, resident males 82.93, SE= 37.29) moved more than females (msd: 23.04, SE=2.67). Nomadic males and females moved more in the wet season (nomadic male slope coefficient: 0.57, SE=0.0004,

p<0.0001; female slope coefficient: 0.14, SE=0.001, p<0.0001), whereas resident males moved more in the dry season (slope coefficient: -0.14, SE=0.0006, p<0.0001).

Landscape-scale habitat selection

At the landscape-scale, all lions selected for habitats with high cover, close to rivers and in flatter terrain (TRI; Fig. 2). All lions also avoided areas of higher intensity of human activity (Fig. 2). Lions showed little landscape-scale selection for distances from human activity in the dry season, however resident males and females selected areas that were closer to human activity in the wet season, while nomadic males selected for areas slightly farther from human activity in the wet season (Fig. 2). While females and nomadic males selected for areas with higher greenness (EVI) regardless of season, resident male selected for areas with lower greenness (Fig. 2).

Fig 2: Landscape-scale habitat selection by females, resident male and nomadic male lions. Slope coefficients associated with landscape-scale habitat selection (RSF) for three classes of lion (females, resident males and nomadic males) in the Ngorongoro Conservation Area, Tanzania. Blue points are habitat covariates and orange points are human-related covariates. Dens_human represents intensity of human activity while Dist_human represents distance to human activity. Coefficients are estimated separately for seasons (wet and dry). All lions selected for habitats with more forest/shrub cover, close to rivers and in flatter terrain (TRI). Females and nomadic males selected for habitats of higher EVI, while resident males selected for lower EVI. All lions avoided areas of higher intensity of human activity. Lions showed little

landscape-scale selection for distances from human activity in the dry season, however resident males and females selected areas closer to human activity in the wet season, while nomadic males selected for areas slightly farther from human activity in the wet season.

Local scale habitat selection

For all three lion categories, a fully parameterized SSF model which included interactions between the percent of cover habitat within a 50m radius and human activity was supported over all other models (S2 Table). UHC plots indicated a well-calibrated and representative model (S2-S4 Figures). The percent of shrub habitat in a 50m radius area was a strong predictor of step-selection for all lion categories (Fig. 3), but relationships varied with season, time of day, and the distance to and intensity of human activity (Figs. 3, 4). At mean levels of distance to and intensity of human activity, all lions were significantly more likely to select locations with more cover during the day compared to at night (Fig. 3). Females more consistently selected for cover overall than did males (Fig. 4), with males, both nomadic and resident, appearing to respond less to cover at higher intensities of human activity during the night and dry season, selecting for less cover at night when moving closer to human activity (Fig. 4, S3 Table, S5 Figure). Males selected significantly for more cover during the wet season, especially when farther from human activity (Figs. 3, 4) while females selected significantly for more cover during the dry season, especially when closer to human activity (Figs. 3, 4). Resident males also selected for more cover during the dry season closer to human activity, but not during the wet season (Fig. 4).

Fig 3: Local-scale habitat selection by females, nomadic males and resident male lions. Slope coefficients associated with local-scale habitat selection (SSF) for three classes of lion (females, nomadic males and resident males) in the Ngorongoro Conservation Area, Tanzania. Blue points are habitat covariates, orange points are human-related covariates. Coefficients are estimated using all data points (top), then separately for seasons (wet and dry) and time of day (night and day). Dens_human represents intensity of human activity while Dist_human represents distance to human activity. The lions' habitat selection strengths varied with season and time of day, with generally less avoidance for humans in the dry season and at night.

Fig 4: Lions' selection for degree of forest/shrub cover when either far or near to humans. Interactions between the distance to human activity (bomas) and local-scale selection (SSF) for forest/shrub cover by female (A), resident male (B) and nomadic male (C) lions. The colors show relationships specific to daytime and nighttime movements, and movements within dry and wet seasons. The panels show step-selection relative to cover far from human activity (left) and closer to human activity (right). All lions selected for more cover farther from human activity, especially during the day and wet season for males and during the day and dry season for females. Closer to human activity, males selected for less cover at night and more cover during the day. Resident males also selected for more cover during the dry season closer to human activity, but not during the wet season. Females showed similar patterns of selection to those seen when farther from human activity, preferring areas with more cover.

Overall, lions were less likely to select locations closer to human activity at mean levels of cover, with this relationship (i.e., avoidance of humans) being significantly stronger during the day for females and male residents (Fig. 3). At mean levels of cover, both resident and nomadic males were significantly less likely to select locations closer to human activity during the wet season while females were slightly more likely to select location closer to human activity, though this result was not statistically significant (Fig. 3). Furthermore, females were significantly more likely to select locations in higher intensities of human activity at mean levels of cover, regardless of season or time of day (Fig. 3). Resident males were significantly more likely to select locations in low intensities of human activity during the day compared to at night at mean levels of cover (Fig. 3).

All lions selected for locations closer to rivers, particularly resident males and females, with this relationship being similar between times of day for all lions and between seasons for nomadic males (Fig. 3). However, females and resident males selected locations closer to rivers more strongly during the wet season compared to the dry season (Fig. 3). All lions were significantly more likely to select locations in lower EVI and TRI, except resident males which showed no significant relationship with EVI (Fig. 3). Relationships with EVI and TRI did not differ significantly between seasons or times of day, except for nomadic males which showed a negative relationship with EVI during the day and a positive relationship at night (Fig. 3). Resident males showed a similarly different relationship with EVI between night and day, but differences were not statistically significant (Fig. 3).

RSS and resistance mapping

RSS was highest for cover for females and resident males (Fig. 5). For nomadic males, the variable with the highest relative selection strength was the interaction between cover and the distance to human activity (Fig. 5). Relative resistance values across the study area were overall higher in the wet season compared to the dry season (Fig. 6). Around the Crater highlands and escarpment (southeastern block of the NCA), our resistance map presented a mosaic of areas of high and low resistance, where although village centers presented high resistance to lion movements, these areas were small and interspersed with patches of low resistance habitat (Fig. 6). The vast short-grass plains across the western part of the NCA has relatively little cover and sparse human settlements and represents an intermediate and homogenous level of resistance for lions (Fig. 6). A portion of the short-grass plains to the southwest of Ndutu is uninhabited by people, thus representing low resistance for lions (Fig. 6). Despite the patchy distribution of low resistance habitats, our analysis did not detect any obvious major barriers to lion movements between the Crater and the neighboring Serengeti NP.

Fig 5: Lions' relative selection strength for habitat variables in the Ngorongoro Conservation Area, Tanzania.

Relative selection strength for variables based on a local scale habitat selection model (SSF) for three classes of lion (females, nomadic males and resident males) in the Ngorongoro Conservation Area, Tanzania. Dens_human represents intensity of human activity while Dist_human represents distance to human activity. Relative selection strength was highest for habitat variable Cover (the percent of forest/shrub cover in a 50m radius) for females and

resident males. For nomadic males, the variable with the highest relative selection strength was the distance to human activity.

Fig 6: Resistance map for dispersing lions across NCA's multiuse landscape

Resistance map for NCA male lions in dispersal mode (nomadic males, not member of an established pride, and without any protected territory) in wet and dry seasons showing major regions, ward centers (circles) and villages (triangles). Relative resistance values, based on local-scale habitat selection model, SSF, are overall higher in the wet season (red = highest resistance/avoidance, yellow = neutral, blue = least resistance/attraction). Relative resistance was generally highest in the human-dominated areas immediately to the west and north of the Ngorongoro Crater, and in steeper terrain. The areas of least resistance to lions occurred in forested corridors just north and south of these human-dominated areas.

Discussion

We analyzed lions' habitat use at two scales to assess the relationship between habitat, the intensity of anthropogenic presence and lion use of a shared landscape. At the landscape scale, lions selected for regions with much cover, close to rivers, flatter terrain, and low intensity of human activity. Greenness (EVI) was also an important factor at the landscape scale, but its effect varied with lion category. At the local scale, while EVI, TRI and distance to rivers were not strongly associated with lion habitat selection, we found that cover, coupled with human activity, was an important potential driver of lion movements, with relationships varying by lion category, season, and time of day. Specifically, we found evidence that lions adjusted their

movements spatially and temporally to behaviorally avoid humans on the landscape. This was most apparent for males, especially nomadic males, which range widely and are most likely to encounter humans on the landscape. As wide-ranging carnivores increasingly inhabit fragmented landscapes under anthropogenic pressure, understanding how they respond to human presence and use suboptimal habitats to disperse between subpopulations will be critical for long-term large carnivore conservation.

Our prediction that human factors would have the largest effect on lions' use of the shared landscape was partially supported. All lion categories showed avoidance of higher intensities of human activity at the landscape scale. At the local scale, based on AIC, we found most support for SSF models that included both human and habitat factors, and their interaction. However, the amount of cover was the most important habitat factor overall at both scales, landscape and local. This is consistent with lions using cover to navigate the landscape, both to hunt prey [38,39] and to avoid dangers, including conflict with humans [59]. At the local scale, cover was especially important for females, possibly because females tend to settle into tight, familiar home ranges, allowing them to identify the best areas for hunting and more easily avoid risky situations. Indeed, although females selected for areas of high greenness (high EVI, an indication of herbivore (lion prey) abundance) across the landscape, they tended to avoid it at the local scale. This may represent female fine-scale behavioral choices to reduce potential conflict with humans in areas where both wild prey and livestock are likely abundant. In contrast, males' habitat selection at the local scale, especially the nomadic males, was not solely dependent on cover, but instead most strongly associated with the interaction between cover and the distance to human activity. Since males move more

widely and faster than females, especially when exploring new, unfamiliar areas [26], they may be unable to identify and exploit cover to the same extent as females. However, when moving through areas with higher intensity of human activity, nomadic males regularly selected for more cover, consistent with an awareness of humans on the landscape and behavioral adaptations for conflict avoidance. We note that since our UHC plots demonstrated good fit for our models [55], we did not consider a non-linear relationship between lion step selection and distance to humans. However, since humans likely represent both a risk (lion hunting) and a benefit (source of livestock) to lions, nonlinearities in this relationship could exist that we have not explored.

All lions avoided areas of high intensity human activity across the landscape and selected for habitats farther from human activity at the local scale. However, this local scale avoidance of humans was lower at night for all lions, consistent with other lion studies in the region [22,59], and during the dry season. This supports our prediction that the response of lions to humans on the landscape will change with time of day and season. Our prediction of higher avoidance of human activity at the landscape scale in the wet season was also supported for females and nomadic males, although resident males responded similarly to humans in both seasons. However, counter to our prediction, male lions (nomadic and resident) tended to avoid humans at the local scale more during the wet season despite there being less intense land use by humans during the wet season when herders spread out given ample pasture and water for livestock. The finding of more avoidance of humans, even at the local scale, during the wet season is consistent with a risk-benefit tradeoff response by the lions [8], where the wet season's high abundance and wider distribution of migratory wild prey species reduces the

need for lions to prey upon riskier livestock. Accordingly, resource scarcity in the dry season likely triggers lions to a higher risk-taking behavior, with decreased human avoidance for access to livestock prey. Indeed, consistent with seasonal fluctuations in wild prey abundance and distribution, females and nomadic males moved more in the wet season and less in the dry season. The observed females' landscape-scale preference for settling closer to human activity during the wet season contradicts the overall higher landscape-scale avoidance of intensity of human activity. However, this result is likely an artifact of fewer humans on portions of the landscape during the wet season, due to avoidance of Malignant Catarrhal Fever, allowing more "available" points in the RSF to have higher distances from humans. Since females maintain smaller and more consistent home ranges throughout the year, being restricted from expanding seasonally due to the presence of neighboring prides, the RSF comparison will estimate proportionally higher use closer to human activity, given the overall larger distances to humans available in the wet season. At the local scale, females did not show a seasonal difference relative to human activity, suggesting that females may be less able to respond to seasonal changes in prey availability to the same extent as males, likely due to their relatively static home ranges. Whether human presence and intensity of human activity across the landscape represents a more consistent factor affecting female habitat use throughout the year, compared to males where it appears more important seasonally, deserves more study.

In the NCA, livestock are guarded constantly, herded to pasture during the day and returned to the relative safety of the bomas at night. To a lion, a boma represents both a danger and a potential food source to which they may be more willing to approach at night when the risk of conflict is reduced [59]. From a behavioral context, when lions were closer to

bomas during the daytime, they selected for more cover, consistent with the behavioral conflict avoidance observed in other carnivores (e.g., brown bear (*Ursus arctos*) [60] and spotted hyena [61]). However, at night, males, especially nomadic males were willing to forgo cover when closer to human activity, seeming to prefer open areas in risky habitats which may represent the necessity of entering open areas to hunt at or near bomas. This may be due to males being generally less fearful of humans, and is consistent with male lions, especially younger nomadic males, tending to be bolder and take more risks than females [5,26,62]. While this may suggest that male lions are more prone to boma attacks than females, we have recorded numerous boma attacks by collared females (unpublished data). However, females consistently chose cover regardless of time of day or proximity to human activity, suggesting that when females attack a boma, they are able to remain under cover. This difference in selection for cover relative to proximity to humans is consistent with females having greater familiarity with their home ranges, while nomadic males, that lack such familiarity with the area, might benefit from greater visibility rather than cover – a risk-avoidance behavior also found in other carnivores [63]. The different habitat use patterns we found show the importance of monitoring lions of different sexes and life stages, to better understand the range of behavioral responses and adaptations to humans for more effective mitigation of conflict.

Our study demonstrates the importance of considering the behaviors of different sexes and behavioral states (i.e., resident/nomad) within different seasons and times of day for assessing wildlife responses to humans on the landscape. Our model of step-selection for nomadic males provides insight into the implications of humans on the landscape for movements of lions through the NCA, and connectivity between the NCA and neighboring lion

populations (i.e., in Serengeti NP). This connectivity may be crucial for the long-term persistence of the lion sub-population in the Ngorongoro Crater, with its long history of close inbreeding [27,64]. Our resistance map suggests that at the current level of human presence and activity the NCA, adequate low resistance habitat is available for lions to facilitate avoidance of humans and successfully move across and/or inhabit the landscape, thereby enabling connectivity between lion subpopulations. Indeed, our resistance map reflects our tracking data and lion observations [65] across the NCA which demonstrates the ability of lions to move through and occupy the multi-use landscape. While our study offers important insights, we identify two major areas where data collection could be improved to provide further value for understanding lion behavior and habitat selection in the NCA. First, we identify the abundance and distribution of natural prey as important factors possibly driving lion movements, however we lacked concrete measures of natural prey abundance across the landscape, instead using season and EVI as proxies. Future studies that measure prey relative abundance across the NCA will be important to improving our understanding of the dynamics between lions, natural prey and humans as lions move through the landscape. Second, we used bomas as a proxy for human and livestock presence, however exact boma locations shift regularly and most livestock depredations happen at pasture which may not be well captured by boma locations and/or EVI. Since livestock represent both an attracting force (as food) and a repelling force (as a source of conflict), more data on livestock relative abundance over space and time would provide a better understanding of direct interactions between lions and livestock, and thus context for human-lion conflict in the NCA.

We show that the human presence on the landscape can affect lion habitat use at multiple scales, with lions balancing risk through behavioral changes, consistent with other recent studies [8,22,59], including preferentially seeking cover habitats and approaching humans only at night. These types of behavioral adaptations have been noted for other carnivores living near humans [60,66] and exemplify the ways in which carnivore behavioral plasticity can promote coexistence with humans. However, humans remain dangerous to carnivores, with retaliatory killings still a major cause of lion mortality in NCA's multiuse landscape [65]. With the human population increasing in the NCA [67] and across the lion's range [15,16], increased levels of conflict can be expected. Strategies to promote human-lion coexistence in the NCA are being implemented, including carnivore conflict mitigation, community engagement, and incentivization programs. Early signs are encouraging that human tolerance for lions in the NCA can be increased, however the extent to which human behavioral plasticity can match lion behavioral choices to mitigate conflict remains to be seen. Our results suggest that external factors such as vegetation productivity and abundance of natural prey on the landscape can affect the potential for conflict through changes in lion habitat use. Environmental changes that negatively affect pasture productivity and/or wild prey abundance (e.g., climate change, drought, degradation of rangelands, invasive species) could be expected to increase levels of human-lion conflict. As human and lion ranges increasingly overlap across fragmented landscapes under changing environmental conditions, a better understanding of factors affecting lion habitat use and behavioral adaptations will be critical for the continued conservation of this iconic species in the NCA and throughout Africa.

Acknowledgements

This research was carried out under permissions granted by the Tanzania Commission for Science and Technology (COSTECH) and Tanzania Wildlife Research Institute (TAWIRI). We would like to thank the Government of Tanzania, TAWIRI, and Ngorongoro Conservation Area Authority for collaboration, and in particular their veterinarians, D. Wambura and the late A. Niaki. We would like to thank S. Capper, A. Estes, C. and L. Foley, and C. Packer for scientific advice on the study and manuscript. This work could not have been done without the KopeLion team, including R. Lelya for major input on lion monitoring and local ecological knowledge, and the NCA communities for guidance and participation.

References:

1. Tucker MA, Böhning-Gaese K, Fagan WF, Fryxell JM, Van Moorter B, Alberts SC, et al. Moving in the Anthropocene: Global reductions in terrestrial mammalian movements. Science. 2018;359: 466–469. doi:10.1126/science.aam9712

2. Lamb CT, Ford AT, McLellan BN, Proctor MF, Mowat G, Ciarniello L, et al. The ecology of human–carnivore coexistence. Proceedings of the National Academy of Sciences. 2020;117: 17876–17883. doi:10.1073/pnas.1922097117

3. Frankham R, Ballou JD, Ralls K, Eldridge M, Dudash MR, Fenster CB, et al. A Practical Guide for Genetic Management of Fragmented Animal and Plant Populations. Oxford University Press; 2019.

4. Gibbs JP. Demography versus habitat fragmentation as determinants of genetic variation in wild populations. Biological Conservation. 2001;100: 15–20. doi:10.1016/S0006-3207(00)00203-2

5. Bonte D, Van Dyck H, Bullock JM, Coulon A, Delgado M, Gibbs M, et al. Costs of dispersal. Biological Reviews. 2012;87: 290–312. doi:10.1111/j.1469-185X.2011.00201.x

6. Pereira P, Alves da Silva A, Alves J, Matos M, Fonseca C. Coexistence of carnivores in a heterogeneous landscape: habitat selection and ecological niches. Ecol Res. 2012;27: 745–753. doi:10.1007/s11284-012-0949-1

7. Delibes M, Ferreras P, Gaona P. Attractive sinks, or how individual behavioural decisions determine source–sink dynamics. Ecology Letters. 2001;4: 401–403. doi:10.1046/j.1461-0248.2001.00254.x

8. Mills KL, Belant JL, Beukes M, Dröge E, Everatt KT, Fyumagwa R, et al. Tradeoffs between resources and risks shape the responses of a large carnivore to human disturbance. Commun Biol. 2023;6: 1–11. doi:10.1038/s42003-023-05321-z

9. Gaynor KM, Hojnowski CE, Carter NH, Brashares JS. The influence of human disturbance on wildlife nocturnality. Science. 2018;360: 1232–1235. doi:10.1126/science.aar7121

10. Nickel BA, Suraci JP, Allen ML, Wilmers CC. Human presence and human footprint have non-equivalent effects on wildlife spatiotemporal habitat use. Biological Conservation. 2020;241: 108383. doi:10.1016/j.biocon.2019.108383

11. Oriol-Cotterill A, Valeix M, Frank LG, Riginos C, Macdonald DW. Landscapes of Coexistence for terrestrial carnivores: the ecological consequences of being downgraded from ultimate to penultimate predator by humans. Oikos. 2015;124: 1263–1273. doi:10.1111/oik.02224

12. Sage AH, Hillis V, Graves RA, Burnham M, Carter NH. Paths of coexistence: Spatially predicting acceptance of grizzly bears along key movement corridors. Biological Conservation. 2022;266: 109468. doi:10.1016/j.biocon.2022.109468

13. Wilkinson CE, McInturff A, Miller JRB, Yovovich V, Gaynor KM, Calhoun K, et al. An ecological framework for contextualizing carnivore–livestock conflict. Conservation Biology. 2020;34: 854–867. doi:10.1111/cobi.13469

14. Loveridge AJ, Valeix M, Elliot NB, Macdonald DW. The landscape of anthropogenic mortality: how African lions respond to spatial variation in risk. Journal of Applied Ecology. 2017;54: 815–825. doi:10.1111/1365-2664.12794

15. Riggio J, Jacobson A, Dollar L, Bauer H, Becker M, Dickman A, et al. The size of savannah Africa: a lion's (Panthera leo) view. Biodivers Conserv. 2013;22: 17–35. doi:10.1007/s10531-012-0381-4

16. Sargent R, Deere NJ, McGowan PJK, Bunnefeld N, Pfeifer M. Room to roam for African lions Panthera leo: a review of the key drivers of lion habitat use and implications for conservation. Mammal Review. 2022;52: 39–51. doi:10.1111/mam.12262

17. Homewood KM, Rodgers WA. Maasailand ecology: pastoralist development and wildlife conservation in Ngorongoro, Tanzania. Maasailand ecology: pastoralist development and wildlife conservation in Ngorongoro, Tanzania. 1991 [cited 7 Aug 2023]. Available: https://www.cabdirect.org/cabdirect/abstract/19921850333

18. Nelson F. Natural conservationists? Evaluating the impact of pastoralist land use practices on Tanzania's wildlife economy. Pastoralism. 2012;2: 15. doi:10.1186/2041-7136-2-15

19. Kissui BM. Livestock predation by lions, leopards, spotted hyenas, and their vulnerability to retaliatory killing in the Maasai steppe, Tanzania. Animal Conservation. 2008;11: 422–432. doi:10.1111/j.1469-1795.2008.00199.x

20. Hazzah L, Borgerhoff Mulder M, Frank L. Lions and Warriors: Social factors underlying declining African lion populations and the effect of incentive-based management in Kenya. Biological Conservation. 2009;142: 2428–2437. doi:10.1016/j.biocon.2009.06.006

21. Ikanda D, Packer C. Ritual vs. retaliatory killing of African lions in the Ngorongoro Conservation Area, Tanzania. Endangered Species Research. 2008;6: 67–74. doi:10.3354/esr00120

22. Suraci JP, Frank LG, Oriol-Cotterill A, Ekwanga S, Williams TM, Wilmers CC. Behavior-specific habitat selection by African lions may promote their persistence in a human-dominated landscape. Ecology. 2019;100: e02644. doi:10.1002/ecy.2644

23. Packer C. The lion: Behavior, ecology, and conservation of an iconic species. Princeton, NJ: Princeton University Press; 2023.

24. Pusey AE, Packer C. The Evolution of Sex-Biased Dispersal in Lions. Behaviour. 1987;101: 275–310. doi:10.1163/156853987X00026

25. Elliot NB, Cushman SA, Loveridge AJ, Mtare G, Macdonald DW. Movements vary according to dispersal stage, group size, and rainfall: the case of the African lion. Ecology. 2014;95: 2860–2869. doi:10.1890/13-1793.1

26. Elliot NB, Cushman SA, Macdonald DW, Loveridge AJ. The devil is in the dispersers: predictions of landscape connectivity change with demography. Journal of Applied Ecology. 2014;51: 1169–1178. doi:10.1111/1365-2664.12282

27. Packer C, Pusey AE, Rowley H, Gilbert DA, Martenson J, O'Brien SJ. Case Study of a Population Bottleneck: Lions of the Ngorongoro Crater. Conservation Biology. 1991;5: 219–230.

28. Århem K. Pastoral man in the garden of Eden: the Maasai of the Ngorongoro conservation area, Tanzania. University of Uppsala: The Scandinavian Institute of African Studies; 1985 pp. 1–123. Available: https://www.diva-portal.org/smash/record.jsf?pid=diva2%3A277704&dswid=-8737

29. Metzger KL, Sinclair ARE, Macfarlane A, Coughenour MB, Ding J. Scales of change in the Greater Serengeti ecosystem. Serengeti IV: Sustaining Biodiversity in a Coupled Human–Natural System. Chicago and London: The University of Chicago Press; 2015. pp. 33–71.

30. Holdo RM, Holt RD, Fryxell JM. Opposing Rainfall and Plant Nutritional Gradients Best Explain the Wildebeest Migration in the Serengeti. The American Naturalist. 2009;173: 431–445. doi:10.1086/597229

31. Homewood K, Kristjanson P, Trench PC. Changing Land Use, Livelihoods and Wildlife Conservation in Maasailand. In: Homewood K, Kristjanson P, Trench PC, editors. Staying Maasai? Livelihoods, Conservation and Development in East African Rangelands. New York, NY: Springer; 2009. pp. 1–42. doi:10.1007/978-0-387-87492-0_1

32. Pennycuick L. Movements of the migratory wildebeest population in the Serengeti area between 1960 and 1973. African Journal of Ecology. 1975;13: 65–87. doi:10.1111/j.1365-2028.1975.tb00124.x

33. Sinclair ARE, Metzger KL, Mduma SAR, Fryxell JM. Serengeti IV: Sustaining Biodiversity in a Coupled Human-Natural System. University of Chicago Press; 2015.

34. Estes RD. The significance of breeding synchrony in the wildebeest. African Journal of Ecology. 1976;14: 135–152. doi:10.1111/j.1365-2028.1976.tb00158.x

35. Cleaveland S, Kusiluka L, Kuwai J, Bell C, Kazwala R. ASSESSING THE IMPACT OF MALIGNANT CATARRHAL FEVER IN NGORONGORO DISTRICT, TANZANIA A study commissioned by the Animal Health Programme, Department for International Development. 2023.

36. Plowright W. Malignant Catarrhal Fever in East Africa: I.—Behaviour of the Virus in Free-living Populations of Blue Wildebeest (Gorgon taurinus taurinus, Burchell). Research in Veterinary Science. 1965;6: 56–68. doi:10.1016/S0034-5288(18)34767-2

37. Song L, Estes AB, Estes LD. A super-ensemble approach to map land cover types with high resolution over data-sparse African savanna landscapes. International Journal of Applied Earth Observation and Geoinformation. 2023;116: 103152. doi:10.1016/j.jag.2022.103152

38. Hopcraft JGC, Sinclair ARE, Packer C. Planning for success: Serengeti lions seek prey accessibility rather than abundance. Journal of Animal Ecology. 2005;74: 559–566. doi:10.1111/j.1365-2656.2005.00955.x

39. Loarie SR, Tambling CJ, Asner GP. Lion hunting behaviour and vegetation structure in an African savanna. Animal Behaviour. 2013;85: 899–906. doi:10.1016/j.anbehav.2013.01.018

40. Riley SJ, DeGloria SD, Elliot R. Index that quantifies topographic heterogeneity. Intermountain Journal of Sciences. 1999;5: 23–27.

41. Mosser A, Fryxell JM, Eberly L, Packer C. Serengeti real estate: density vs. fitness-based indicators of lion habitat quality. Ecology Letters. 2009;12: 1050–1060. doi:10.1111/j.1461-0248.2009.01359.x

42. Maliti H, von Hagen C, Hopcraft G. Rivers and small drainages from Greater Serengeti-Mara Ecosystem digitized from 1:50,000 map sheets. In: SERENGETI GIS & DATA [Internet]. 2008 [cited 7 Dec 2022]. Available: https://serengetidata.weebly.com/rivers-and-lakes.html

43. Lehner B, Verdin K, Jarvis A. New Global Hydrography Derived From Spaceborne Elevation Data. Eos, Transactions American Geophysical Union. 2008;89: 93–94. doi:10.1029/2008EO100001

44. McGarigal K, Wan HY, Zeller KA, Timm BC, Cushman SA. Multi-scale habitat selection modeling: a review and outlook. Landscape Ecol. 2016;31: 1161–1175. doi:10.1007/s10980-016-0374-x

45. Laforge MP, Brook RK, van Beest FM, Bayne EM, McLoughlin PD. Grain-dependent functional responses in habitat selection. Landscape Ecol. 2016;31: 855–863. doi:10.1007/s10980-015-0298-x

46. Singh NJ, Allen AM, Ericsson G. Quantifying Migration Behaviour Using Net Squared Displacement Approach: Clarifications and Caveats. Hays G, editor. PLoS ONE. 2016;11: e0149594. doi:10.1371/journal.pone.0149594

47. Slade NA, Swihart RK. Home Range Indices for the Hispid Cotton Rat (Sigmodon hispidus) in Northeastern Kansas. Journal of Mammalogy. 1983;64: 580–590. doi:10.2307/1380513

48. Thieurmel B, Elmarhraoui A. Package ‘suncalc.’ 2022.

49. Signer J, Fieberg J, Avgar T. Animal movement tools (amt): R package for managing tracking data and conducting habitat selection analyses. Ecology and Evolution. 2019;9: 880–890. doi:10.1002/ece3.4823

50. R Development Core Team. R: a language and environment for statistical computing. R Foundation for Statistical Computing, Vienna. 2019. Available: https://www.r-project.org/

51. Brooks M E, Kristensen K, Van Benthem KJ, Magnusson A, Berg C W, Nielsen A, et al. glmmTMB Balances Speed and Flexibility Among Packages for Zero-inflated Generalized Linear Mixed Modeling. The R Journal. 2017;9: 378. doi:10.32614/RJ-2017-066

52. Muff S, Signer J, Fieberg J. Accounting for individual-specific variation in habitat-selection studies: Efficient estimation of mixed-effects models using Bayesian or frequentist computation. Journal of Animal Ecology. 2020;89: 80–92. doi:10.1111/1365-2656.13087

53. Avgar T, Potts JR, Lewis MA, Boyce MS. Integrated step selection analysis: bridging the gap between resource selection and animal movement. Methods in Ecology and Evolution. 2016;7: 619–630. doi:10.1111/2041-210X.12528

54. Fieberg J, Signer J, Smith B, Avgar T. A ‘How to’ guide for interpreting parameters in habitat-selection analyses. Journal of Animal Ecology. 2021;90: 1027–1043. doi:10.1111/1365-2656.13441

55. Fieberg JR, Forester JD, Street GM, Johnson DH, ArchMiller AA, Matthiopoulos J. Used-habitat calibration plots: a new procedure for validating species distribution, resource selection, and step-selection models. Ecography. 2018;41: 737–752. doi:10.1111/ecog.03123

56. Avgar T, Lele SR, Keim JL, Boyce MS. Relative Selection Strength: Quantifying effect size in habitat- and step-selection inference. Ecology and Evolution. 2017;7: 5322–5330. doi:10.1002/ece3.3122

57. Gelmi-Candusso TA, Chin AT, Thompson CA, McLaren AAD, Wheeldon TJ, Patterson BR, et al. Dynamic connectivity assessment for a terrestrial predator in a metropolitan region. Frontiers in Ecology and the Environment. 2023;In Press.

58. Burnham KP, Anderson DR. Model Selection and Multimodel Inference: A Practical Information-Theoretic Approach. New York, New York, USA: Springer; 2002.

59. Oriol-Cotterill A, Macdonald DW, Valeix M, Ekwanga S, Frank LG. Spatiotemporal patterns of lion space use in a human-dominated landscape. Animal Behaviour. 2015;101: 27–39. doi:10.1016/j.anbehav.2014.11.020

60. Ordiz A, Støen O-G, Delibes M, Swenson JE. Predators or prey? Spatio-temporal discrimination of human-derived risk by brown bears. Oecologia. 2011;166: 59–67. doi:10.1007/s00442-011-1920-5

61. Boydston EE, Kapheim KM, Watts HE, Szykman M, Holekamp KE. Altered behaviour in spotted hyenas associated with increased human activity. Animal Conservation. 2003;6: 207–219. doi:10.1017/S1367943003003263

62. Patterson BD, Kasiki SM, Selempo E, Kays RW. Livestock predation by lions (Panthera leo) and other carnivores on ranches neighboring Tsavo National ParkS, Kenya. Biological Conservation. 2004;119: 507–516. doi:10.1016/j.biocon.2004.01.013

63. Davies AB, Tambling CJ, Marneweck DG, Ranc N, Druce DJ, Cromsigt JPGM, et al. Spatial heterogeneity facilitates carnivore coexistence. Ecology. 2021;102: e03319. doi:10.1002/ecy.3319

64. Kissui BM, Packer C. Top-down population regulation of a top predator: lions in the Ngorongoro Crater. Proc Biol Sci. 2004;271: 1867–1874. doi:10.1098/rspb.2004.2797

65. Jansson I. Progress Report 2023_Balancing Pastoralism & Lion Ngorongoro. Ngorongoro, Arusha, Tanzania: Research project: Balancing Pastoralist Livelihoods and Wildlife Management; 2023 May pp. 1–16.

66. Leighton GRM, Bishop JM, Merondun J, Winterton DJ, O'Riain MJ, Serieys LEK. Hiding in plain sight: risk mitigation by a cryptic carnivore foraging at the urban edge. Animal Conservation. 2022;25: 244–258. doi:10.1111/acv.12732

67. NBS. 2022 Population and Housing Census - Administrative units Population Distribution and Age and Sex Distribution Reports. Ministry of Finance and Planning, National Bureau of Statistics, United Republic of Tanzania; 2022. Available: https://www.nbs.go.tz/nbs/takwimu/Census2022/Administrative_units_Population_Distribution_Report_Tanzania_volume1a.pdf

Supporting information captions

S1 Table: Collared lions, their category, and collaring periods

S2 Table: Model selection results for step-selection functions for female, resident male, and nomadic male lions in Ngorongoro Conservation Area, Tanzania between 2012-2023. Dens_human represents intensity of human activity while Dist_human represents distance to human activity.

S3 Table: Test of the best radius over which to calculate the intensity of human activity on the landscape (i.e., boma density) for each lion category in Ngorongoro Conservation Area, Tanzania.

S1 Figure: Study area in the Ngorongoro Conservation Area showing the distribution of rivers (A) and bomas (B). The green polygon is the Ngorongoro Crater.

S2 Figure: UHC plots for the top step-selection model for nomadic male lions in Ngorongoro Conservation Area, Tanzania. The observed distribution of each covariate at the presence points in the test dataset is given by the solid black lines, with associated 95% simulation envelope in gray. The available habitat across the landscape relative to each covariate is given in the dashed red line. Models were well-calibrated, with the observed distributions (solid black lines) falling predominantly within the gray simulation envelopes. Dens_human represents intensity of human activity while Dist_human represents distance to human activity.

S3 Figure: UHC plots for the top step-selection model for resident male lions in Ngorongoro Conservation Area, Tanzania. The observed distribution of each covariate at the presence points in the test dataset is given by the solid black lines, with associated 95% simulation envelope in gray. The available habitat across the landscape relative to each covariate is given in the dashed red line. Models were well-calibrated, with the observed distributions (solid black lines) falling predominantly within the gray simulation envelopes. Dens_human represents intensity of human activity while Dist_human represents distance to human activity.

S4 Figure: UHC plots for the top step-selection model for female lions in Ngorongoro Conservation Area, Tanzania. The observed distribution of each covariate at the presence points in the test dataset is given by the solid black lines, with associated 95% simulation envelope in gray. The available habitat across the landscape relative to each covariate is given in the dashed red line. Models were well-calibrated, with the observed distributions (solid black lines) falling predominantly within the gray simulation envelopes. Dens_human represents intensity of human activity while Dist_human represents distance to human activity

S5 Figure: Interactions between the intensity of human activity (i.e., boma density) and lion selection for forest/shrub cover for female (A), resident male (B), nomadic male (C) lions. The colors show relationships specific to daytime and nighttime, and within dry and wet seasons movements, respectively. The panels show step-selection relative to forest/shrub cover in low (left) and high (right) intensities of human activity. Lions selected for more forest/shrub cover regardless of the intensity of human activity, but especially during the day and wet season for males and during the day and dry season for females. In higher intensities of human activity, males tended to respond less to forest/shrub cover on the landscape than at lower intensities of human activity where they selected slightly more for forest/shrub cover, especially during the night and dry season.

Fig1

Click here to access/download;Figure;Fig1.tiff

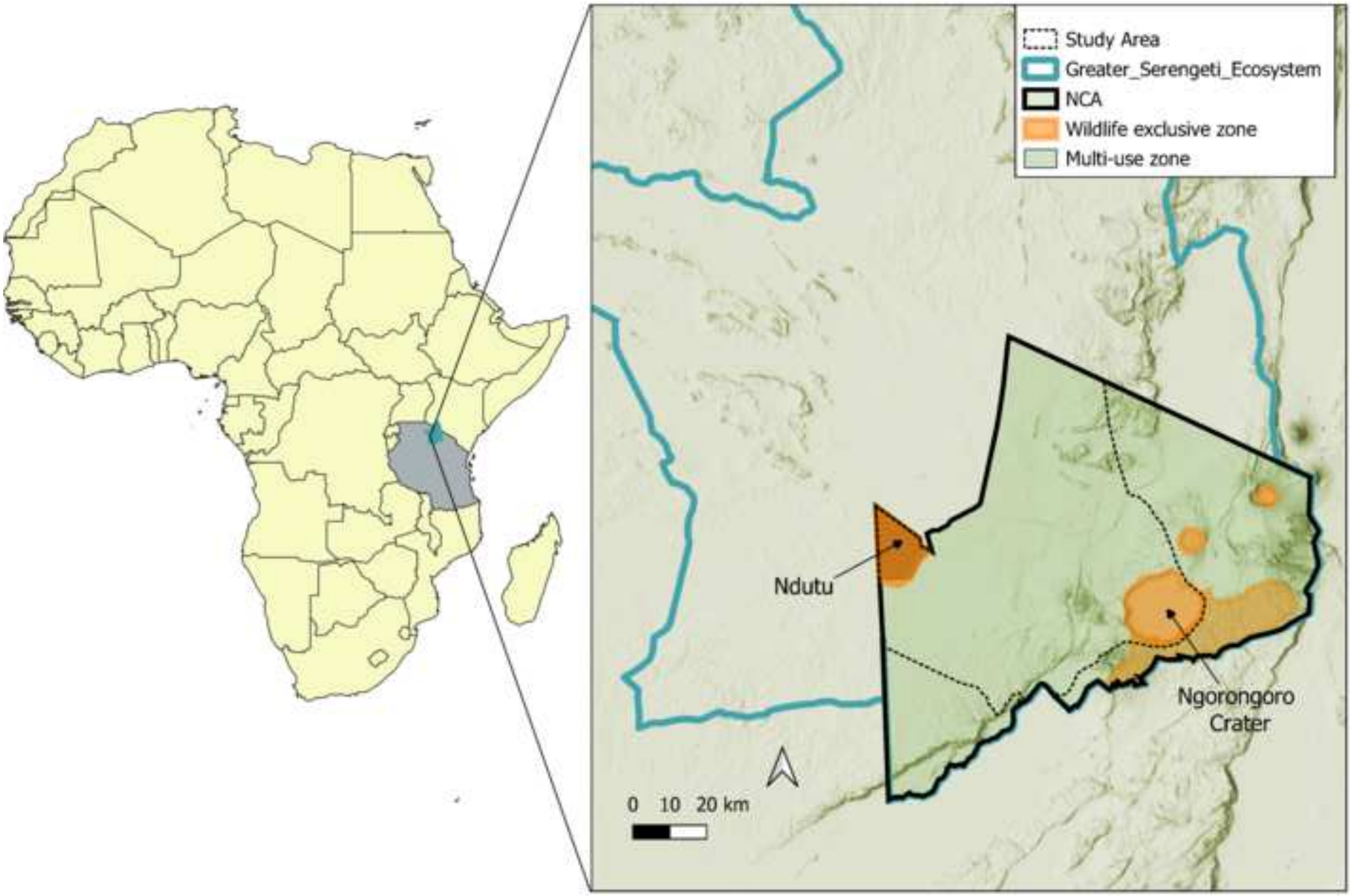

Fig2

Click here to access/download;Figure;Fig2.tiff

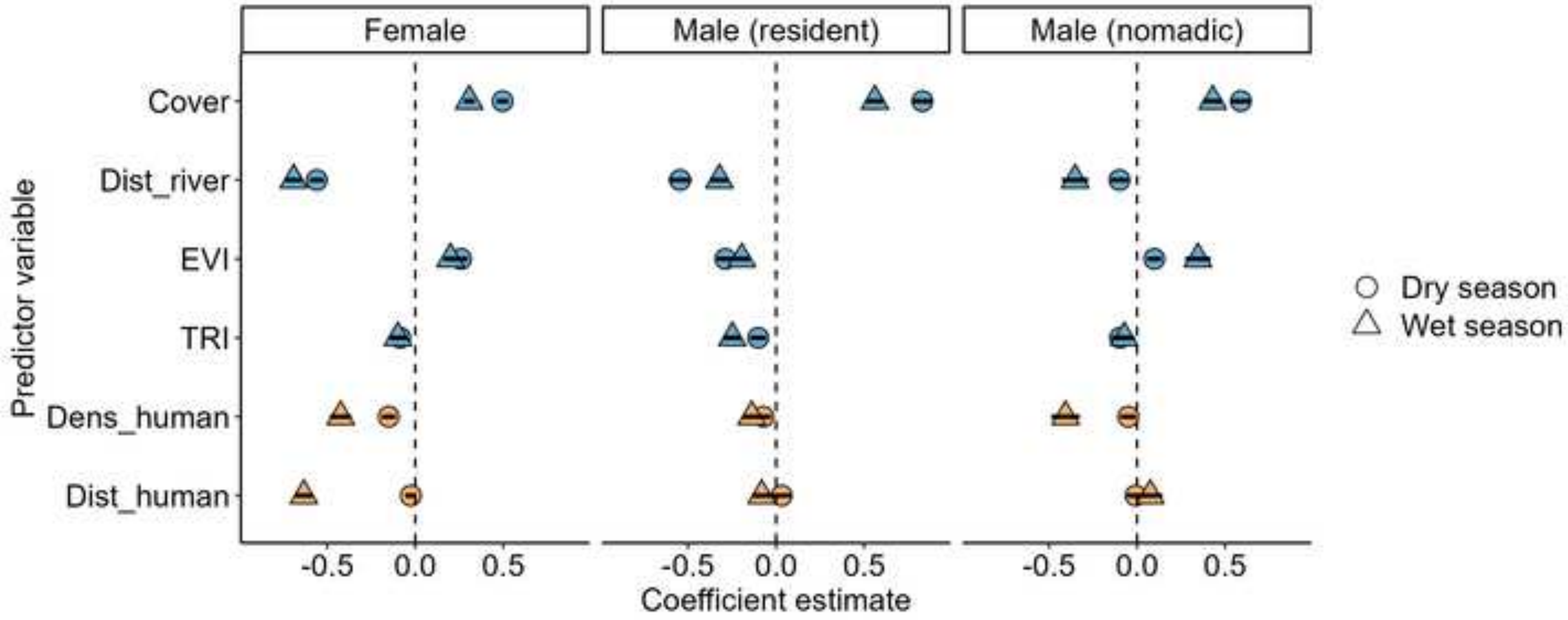

Fig3

Click here to access/download;Figure;Fig3.tiff

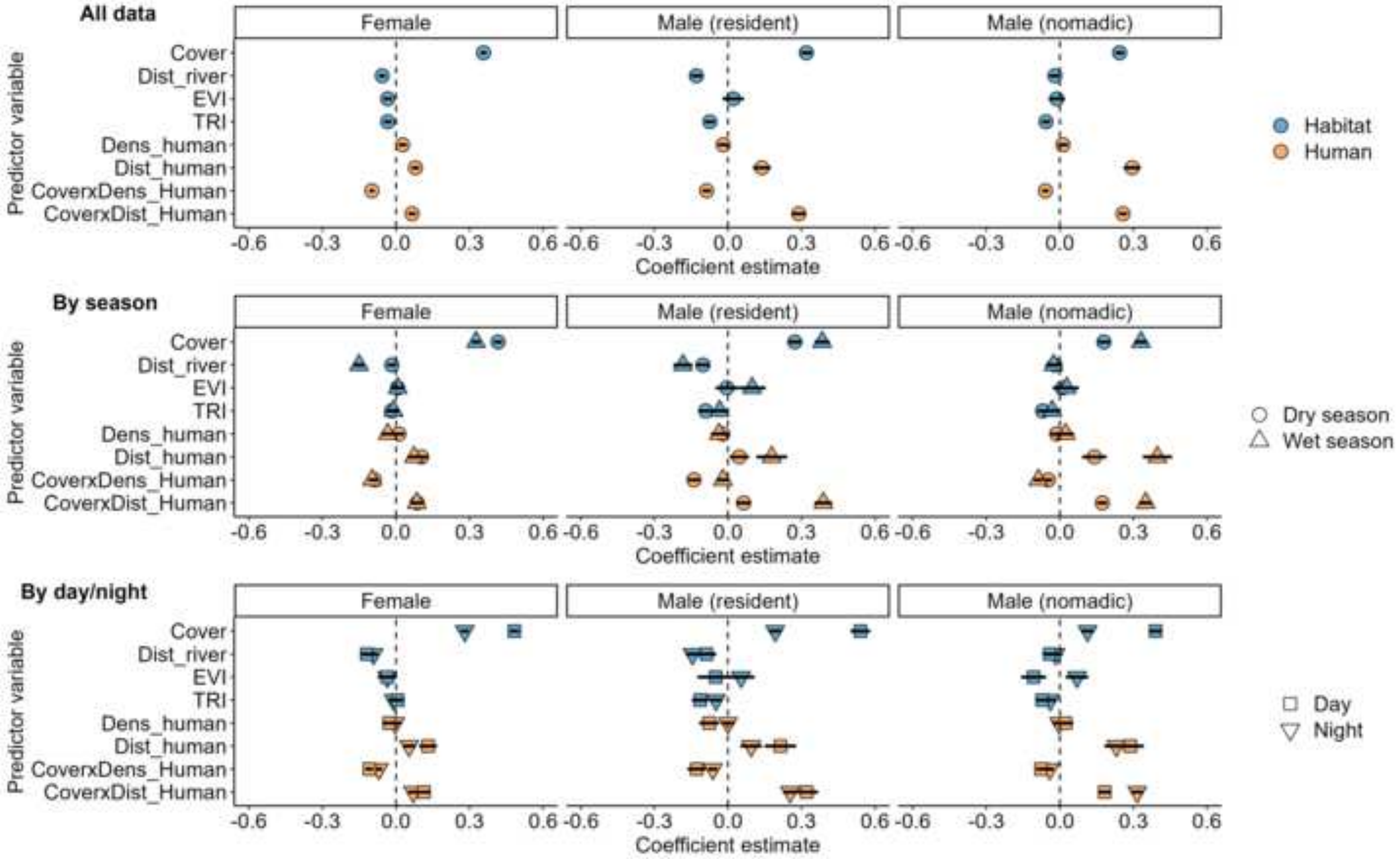

Click here to access/download;Figure;Fig4.tiff

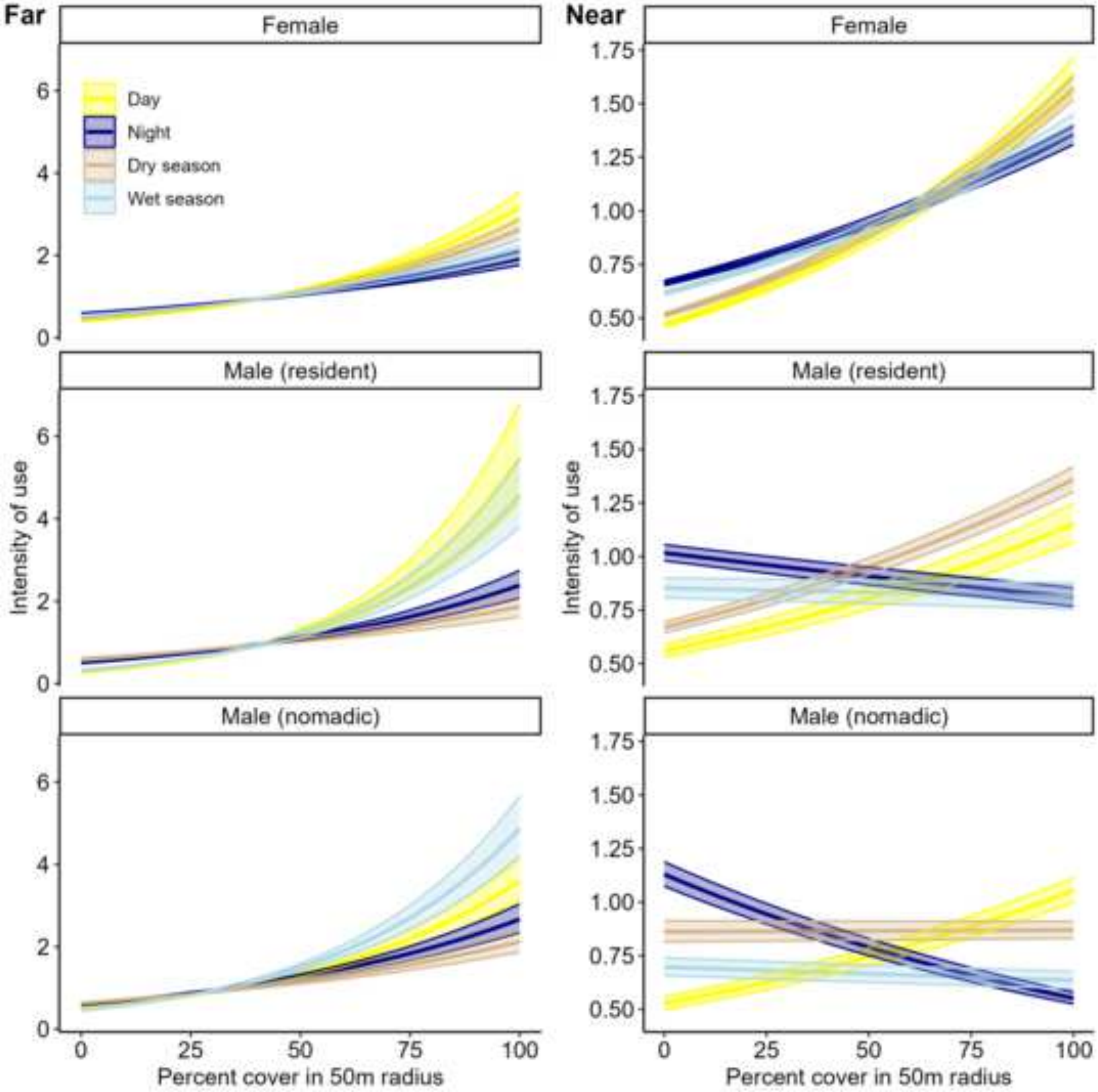

Click here to access/download;Figure;Fig5.tiff

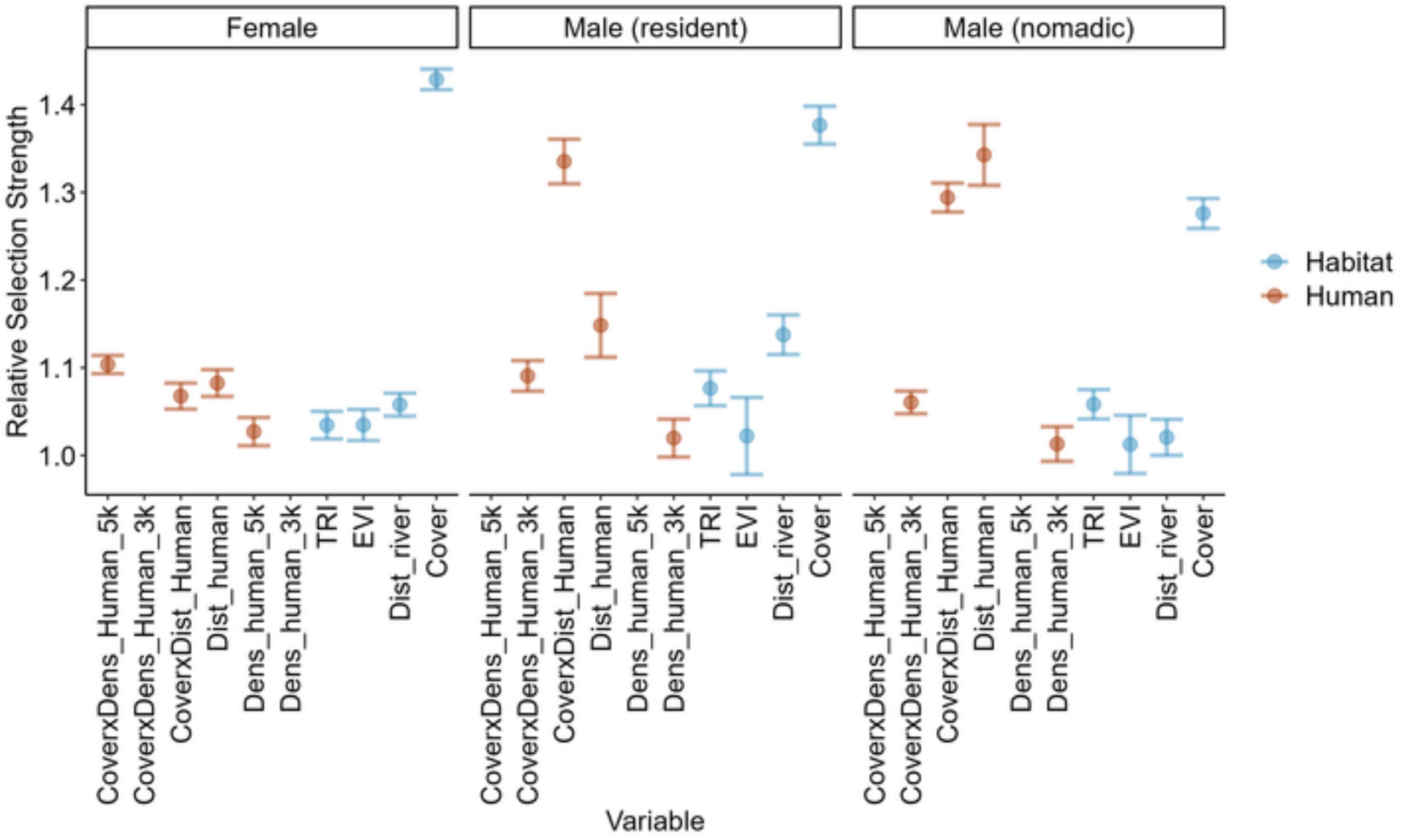

Fig6

Click here to access/download;Figure;Fig6.tiff

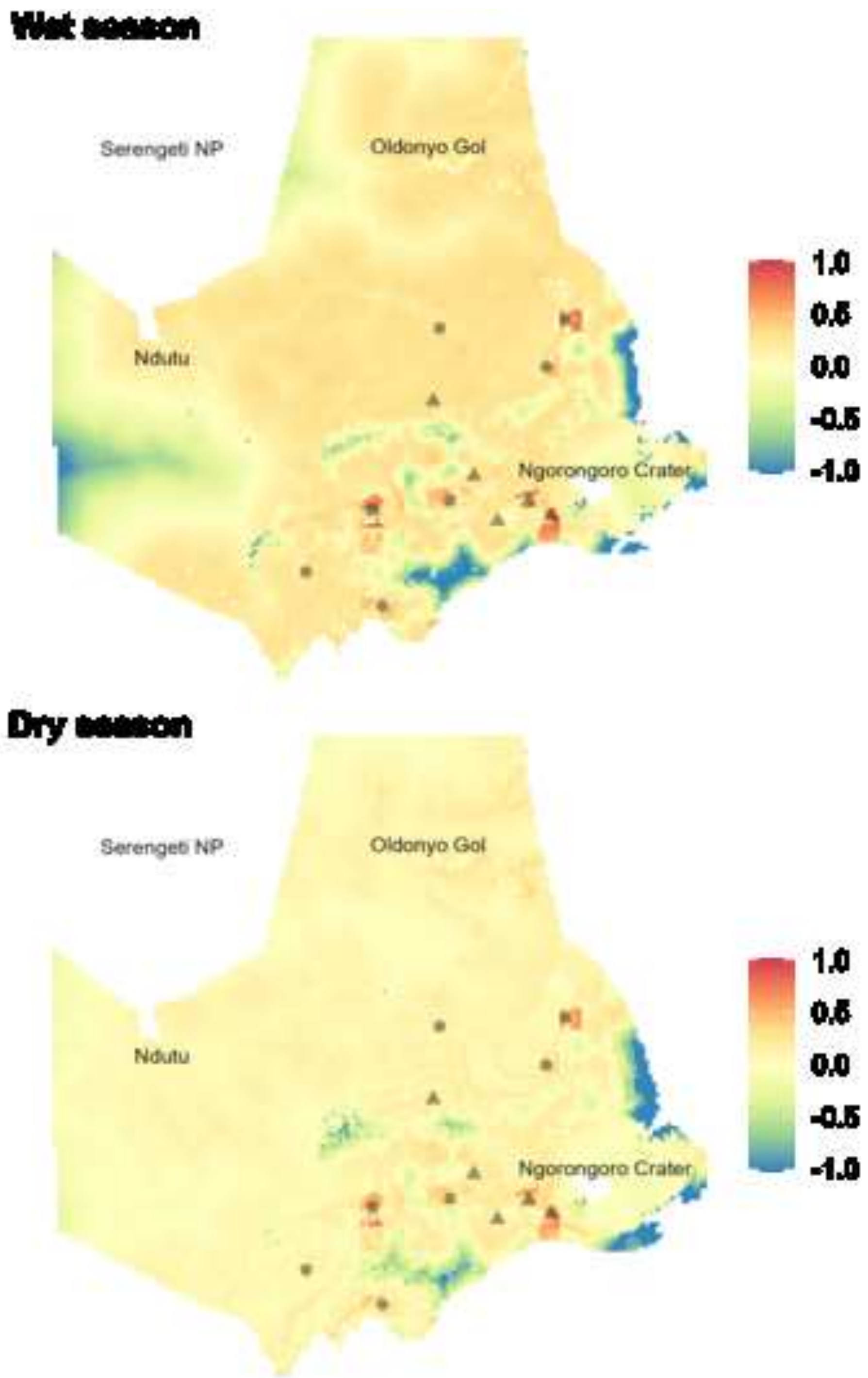

Click here to access/download
Supporting Information
Supporting
information_Jansson_et_al_lion_HabSel.docx
