# Supplementary material for: Coexistence from a lion’s perspective: Movements and habitat selection by African lions (Panthera leo) across a multi-use landscape
Source: PLoS One. 2024 Oct 3;19(10):e0311178. doi: 10.1371/journal.pone.0311178 (PMC11449311; doi:10.1371/journal.pone.0311178)
Supplement: Attachment — Submitted filename: Response to reviewers_PONE-D-24-04716_2.docx [file pone.0311178.s016.docx]

**Response to reviewers_PONE-D-24-04716**

**Editor comments:**

When submitting your revision, we need you to address these additional requirements.

1. Please ensure that your manuscript meets PLOS ONE's style requirements, including those for file naming. The PLOS ONE style templates can be found at

<https://journals.plos.org/plosone/s/file?id=wjVg/PLOSOne_formatting_sample_main_body.pdf>

[ht](https://journals.plos.org/plosone/s/file?id=wjVg/PLOSOne_formatting_sample_main_body.pdf)[tps://journals.plos.org/plosone/s/file?id=ba62/PLOSOne_formatting_sample_title_authors_affiliations.pdf](https://journals.plos.org/plosone/s/file?id=ba62/PLOSOne_formatting_sample_title_authors_affiliations.pdf)

Author response: Thanks for pointing this out. We have followed the instructions and made corrections accordingly.

2. When completing the data availability statement of the submission form, you indicated that you will make your data available on acceptance. We strongly recommend all authors decide on a data sharing plan before acceptance, as the process can be lengthy and hold up publication timelines. Please note that, though access restrictions are acceptable now, your entire data will need to be made freely accessible if your manuscript is accepted for publication. This policy applies to all data except where public deposition would breach compliance with the protocol approved by your research ethics board. If you are unable to adhere to our open data policy, please kindly revise your statement to explain your reasoning and we will seek the editor's input on an exemption. Please be assured that, once you have provided your new statement, the assessment of your exemption will not hold up the peer review process.

Author response: Data are now publicly accessible via this link: <https://doi.org/doi:10.5061/dryad.j6q573nnb>

3. Please include your full ethics statement in the ‘Methods’ section of your manuscript file. In your statement, please include the full name of the IRB or ethics committee who approved or waived your study, as well as whether or not you obtained informed written or verbal consent. If consent was waived for your study, please include this information in your statement as well.

Author response: Thanks for pointing this out. We have added an *Ethical statement* subsection under Methods. This section reads (line 186-222):

“All research, fieldwork and data collection, including animal-handling to deploy collars on lions, complied with the Tanzania Wildlife Research Institute (Conduct of Wildlife Research) Regulations (Tanzania Wildlife Research Institute, 2020), and was carried out under the yearly renewed research permits granted to IJ with No’s 2012-73-ER-90-15, 2013-147-NA-90-15, 2014-159-ER-2007-15, 2015-125-NA-2014-1165, 2016-229-NA-2014-165, 2017-243-NA-2007-15, 2018-362-NA-2014-165, 2019-341-NA-2006-79, 2020-256-NA-2019-065, 2021-548-NA-2019-065, 2022-771-NA-2019-068, and 2023-793-ER-2019-068 by the Tanzania Commission for Science and Technology (COSTECH; Dar es Salaam, Tanzania; rclearance@costech.or.tz) and Tanzania Wildlife Research Institute (TAWIRI; Arusha, Tanzania; researchclearance@tawiri.or.tz). A further permission to deploy collars was granted by NCA Authority, in letters No. NCAA/D/240/VOL.XXI/78, date 30/10/2012, to collar up to six lions simultaneously, and No. BD/158/711/01'E'/54, date 14/12/2021, permission renewed to collar up to eight lions simultaneously (NCAA; Ngorongoro Crater, Arusha, Tanzania; cc@ncaa.go.tz).

In compliance with Tanzanian law, all lion captures and immobilizations for deployment or replacements of collars were performed by a NCA Authority or TAWIRI veterinarian. All collars featured a remote drop-off function, which could be activated via a timer or radio-command using a release transmitter (VECTRONIC Aerospace, 2024). Therefore, re-capture of the lions was not necessary for collar removals. Collars were removed if they malfunctioned, batteries ran low, or the purpose for collaring that animal had been fulfilled.

Lions in NCA community lands are few and elusive, hence finding and capturing targeted individuals to deploy collars is challenging and done opportunistically, mainly at night, following observations of lions in the area. To attract lions closer to the vehicle for immobilization, we used a speaker (model Krakatoa, by FoxPro, USA) to broadcast a high-volume recording of feeding hyenas, a bleating buffalo calf, or the roars of a lion. To capture the lions, they were chemically immobilized with a drug mixture providing sedative, tranquilizing, and anesthetic effects (e.g., Zoletil and medetomidine), administered via a dart shot from a CO2-powered dart-gun (Dan-Inject or Pneu-Dart) from a vehicle at a distance of 10-20 meters, following protocols described in (Kock and Burroughs, 2012). Sedation time was typically less than one hour, during which time the veterinarian and the researcher monitored body conditions closely (breathing, temperature, circulation). The collars were fitted by an experienced field researcher supervised by the veterinarian. Afterwards we remained with the lion to closely monitor it until alert and deemed safe and well recovered after the immobilization.

Weighing approximately 1,200 g, the collars represented 1% or less of an adult lions’ body weight and well within recommended limits (REF Wilson 2021). We closely monitored all collared individuals through visual observations and field signs, and via daily checks of their movements as transmitted by the collars. We were observant of any signs of distress or negative short- or long-term impacts from the collars, including effects on reproduction, hunting and feeding behaviors, and social interactions. No apparent side effects from the collars were detected.”

4. We note that Figures 1 and 6 in your submission contain [map/satellite] images which may be copyrighted. All PLOS content is published under the Creative Commons Attribution License (CC BY 4.0), which means that the manuscript, images, and Supporting Information files will be freely available online, and any third party is permitted to access, download, copy, distribute, and use these materials in any way, even commercially, with proper attribution. For these reasons, we cannot publish previously copyrighted maps or satellite images created using proprietary data, such as Google software (Google Maps, Street View, and Earth). For more information, see our copyright guidelines: <http://journals.plos.org/plosone/s/licenses-and-copyright>.

Author response: For **Figure 1**, we have replaced the basemap to one that is open-access/use and added the following to the figure legend (line 146): “Basemap reprinted from ESA WorldCover project 2020 under a CC BY 4.0 license”. **Figure 6** does not contain any satellite imagery; its contents were created solely by the authors. The contents of Figure 6 include model predictions of our SSF model, generated using a grid of remotely sensed points gathered solely by the authors, using the covariates described in the Methods of the MS. For model prediction, points were generated within the bounds of the study area, which was delineated solely by the authors using Google Earth (border shapefile). This creates the background of the map. The points and labels on the map were also generated solely by the authors using Google Earth. There is no imagery contained within this Figure that was generated by any source other than the authors.

5. First I want to apologize for the time it took to provide a decision on this manuscript, as it was challenging to find reviewers for it. The manuscript addresses an important topic and uses well-established techniques to accomplish its stated goals. The reviewers are generally supportive of the manuscript proceeding to publication, provided that some relatively major revisions are made, most substantively clarifying methodology and acknowledging sample sizes used. I also agree with Reviewer 1 that some of the figures are redundant in the main manuscript because they present relatively similar information.

Author response: Thanks, we are both thrilled and relieved to hear back from you. We have addressed all of the reviewer comments below. These comments are a great help for us to improve on the manuscript.

6. In addition to their comments, please better clarify the classification of male lions in nomadic vs. resident - what thresholds were used to assign the individuals to one of these categories? E.g. how many days minimum did a male lion need to spend with a pride of females for it to be classified as resident? What type of interactions did the male need to have with the females and how did the different criteria factor in the classification? For example, how does a male that spent 30 days with a group of females but did not mate with them compare to a male that spent 3 days with the female but mated with them? Having clearly defined criteria would help not only this study but also classifications in future studies by other authors.

Author response: A good point to have this better clarified. The amended text now include how we used observations of social interactions for our definitions, and the text now reads (lines 284-292): “To define a male lion's social status, we used the same criteria as in our long-term detailed lion monitoring with direct observations and individual recognition (Packer, 2023). A male was considered nomadic when he departed from his natal pride (usually by 2-3 years of age), and/or when he ceased to be the resident male of a pride. A male was defined as a resident when consistently observed with a pride, actively engaging with the females and siring offspring. Resident males tend to spend most of their time with the pride (or among multiple prides if resident in several simultaneously), especially during the takeover and establishment phase, and when they have young cubs (Packer, 2023; Pusey and Packer, 1987). This consistent behavior, coupled with our daily monitoring surveys and movement data from collared lions, enabled us to characterize male status with confidence.”

7. In the Methods you describe that local community members collected spatial data on water availability (river, springs, dams etc.). Please clarify if all these data were included in the modelling procedure. It appears that only distance to river was used for modelling, why not use all data that were collected? Especially given that you state rivers tend to be seasonal, whereas presumably the point water features are mostly permanent year-round.

Author response: Yes, we did include these “water points” in the preliminary analyses – but as they were highly correlated with the boma locations, we removed them. This appears in the text in the first paragraph of the Methods (lines 266-268): “However, preliminary tests indicated that distance to the nearest water point and distance to human activity were highly correlated (0.82), thus we selected only one variable, distance to human activity, for use in our models.” To avoid confusion, we have removed “water points” from Table 1.

8. There is a statement in the manuscript that in lion populations males are the ones that are important for connectivity, I would suggest nuancing this statement as females can also disperse although generally shorter distances. I am not suggesting running the resistance analysis on females also, but I do think that it would be important to mention in the text that females may also play a role in connectivity although perhaps less prominent than males.

Author response: Thanks, that is indeed true. We have amended this to read (Lines 85-86): "Females lions tend to be philopatric and territorial, typically settling adjacent to their natal area if they disperse (Curry et al., 2019; Dolrenry et al., 2014; VanderWaal et al., 2009). In contrast, most males depart from their natal prides at maturity..."

Further, (Lines 351-353) "Since dispersal between lion sub-populations is typically done by males (Curry et al., 2019; Dolrenry et al., 2014) who tend to make initial long-distance movements after leaving their natal pride (Elliot et al., 2014), and sometimes throughout their lives (Packer, 2023), we based our resistance mapping solely on nomadic males."

**Reviewer #1**

9. I have read the manuscript entitled “Coexistence from a lion’s perspective: movements and habitat selection by African lions (Panthera leo) across a multi-use landscape”. The study presents an analysis of lion habitat selection at two scales (landscape and local) in the Ngorongoro Conservation Area, as a function of human activity, in a conservation context aiming at reduce human-lion conflict. The manuscript is interesting and well written, the habitat selection methods are correctly applied, and I think it deserves to be published, despite the small sample size in each lion category (the authors can acknowledge this point in the discussion).

Author response: Thank you! We have added an acknowledgement of sample size to the discussion (Lines 563-567), “While our study offers important insights, we note that our sample size is small, with 2-3 key individuals dominating the dataset for each lion category. While small sample sizes are typical of wildlife tracking studies, especially for large carnivores, our conclusions could have been influenced if those individuals were not representative of the rest of the population.” In addition, we have added the proportion of location data that each lion contributed, overall and within its category in the S1 Table.

I think the method section deserves some clarifications, see my numerous minor comments along these lines below. Specifically, the authors should specify whether they scaled their continuous variables before running the SSF because of a risk of statistical problems for subsequent analyses, and justify why the resistance map varied between -1 and 1 (I don’t understand how is it possible with the current justification). I also strongly recommend to delete the Figure 5, which is redundant to Figure 3A and very misleading. To improve the general clarity of the manuscript, I suggest to improve figure presentation by shading non-significant results and facilitate a quick interpretation. In addition, the discussion is a little long and could be shortened and structured with sub-headings.

Author response: Thank you for good and valid evaluations and comments. We have removed Figure 5, shortened the Discussion and added the suggested shading to Figures 2 and 3.

10. L27. Replace « adaptations » by « adjustments ».

Author response: This has been done.

11. L131. Correct for “12 persons/km²”.

Author response: This has been done.

12. L280. I don’t think you run RSF and SSF with suncalc package. Please reformulate the sentence.

Author response: We have rewritten this to read (lines 303-304): “We separated day- and night-time positions using the R package suncalc”

13. L284. Refer to the logistic regression in the section.

Author response: This has been added.

14. L293. Probably a mistake formulation here. There is no strata in a RSF. Muff et al. also recommend to use random slope coefficients to account for inter-individual variability in habitat selection, and weighted availability.

Author response: Thank you, the references to strata was indeed a typo here and has been removed. We also appreciate the recommendation to use random slopes in our models and a weighted likelihood for the RSF, we have implemented these changes and updated the methods to reflect that. As would be expected, this has decreased coefficient confidence for many relationships, and we have updated the results and discussion to reflect those changes.

15. L298-300. The SSF definition is almost not understandable. I suggest something like “For SSF modelling, each observed step is paired with X random steps, resulting in a stratified dataset.”

Author response: Thank you, we have made the suggested change. This sentence now reads (Lines 321-323): “The available locations for SSF modelling are conditioned on the last step taken by the animal, meaning each observed step is paired with some number of random steps, resulting in a stratified dataset [52].”

16. L340. You have not mentioned that you scaled the continuous variables before adjusting the SSF. You cannot apply the regression coefficients to the new scaled variable if the coefficients were previously estimated using an unscaled variable.

Author response: Thank you for catching that! We did indeed center and scale prior to analysis and have added a sentence to that effect (Line 270): “All continuous variables were centered and scaled prior to analysis.”

17. L342-349. And what about the day/night differences?

Author response: We did not assess day/night differences in resistance, instead focusing on seasonal differences (thus averaging over day/night). We have clarified that in the text (Line 367-368): “For simplicity, we did not model resistance separately by night/day.”

18. L370. “In lieu of…” ? Maybe reformulate for “In absence of…”

Author response: We have made the suggested change.

19. L372-373. Add the distance unit.

Author response: This has been added

20. L372-376. Add a Table presenting the regression summary in Appendix. Without information on the reference category for each categorical covariable, it is not possible to evaluate this part.

Author response: We have added S3 Table as the reviewer suggested.

21. L388. What does the error bar represent?

Author response: We have clarified this by adding (line 403): “Slope coefficients (points) and 95% confidence intervals (lines)”.

22. L393-399. Delete.

Author response: We prefer to have our figures stand alone, which includes, in our opinion, some high-level interpretation of the results. However, if the editor feels strongly, we will remove.

23. L419. What does the error bar represent?

Author response: We have clarified this by adding (line 443): “Slope coefficients (points) and 95% confidence intervals (lines)”.

24. L425-426. Delete.

Author response: We prefer to have our figures stand alone, which includes, in our opinion, some high-level interpretation of the results. However, if the editor feels strongly, we will remove.

25. L432-433. Specify the chosen distance.

Author response: We have clarified those distances as 500m (near) and 2km (far) in the figure legends (Fig 4 and 5).

26. L433-438. Delete.

Author response: We prefer to have our figures stand alone, which includes, in our opinion, some high-level interpretation of the results. However, if the editor feels strongly, we will remove.

27. L442. Specify the value of the mean levels of cover (to the reader have a better idea of the local conditions).

Author response: This has been added (30.1%) (Line 420)

28. L442-444. This results is also true for dry season. Unless you wish to state “both resident and nomadic males were significantly less likely to select locations closer to human activity during the wet season than dry season“. Authors should be clearer in their presentation of the results, specifying the basis for comparison. Verify also the rest of the manuscript.

Author response: Thank you. Although this sentence has been removed in light of the new results, we have made an effort throughout the Results to make sure we are specifying the basis for each comparison.

29. L458-459. Results are already complex to follow due to crossing information on season, time of day and sex, and a little long. I suggest to delete this sentence (and maybe some others) to reduce a bit. To make it easier to read, I also suggest shading non-significant results in grey on the figures 2 and 3.

Author response: This sentence has been deleted, and we have worked through the text in the result section to be more concise and easier to follow. We have greyed-out non-signficant values in Figures 2 and 3 as suggested by the reviewer.

30. L462. I suggest to delete the Figure 5. Figure 5 is redundant with Figure 3A, although it shows the exponential of the estimates. In addition, all the explanations in this figure are redundant with the previous results section. You also use RSS to calculate map resistance only for nomadic males and at both seasons, but Figure 5 shows the results for the 3 lion categories without seasonal distinction, which is very misleading.

Author response: We have taken the reviewer’s advice and removed Figure 5 from the main text. We have retained it in the Supplemental Materials because we find it easier to read quickly and may be preferable to a general audience.

31. L481-484. Delete.

Author response: We prefer to have our figures stand alone, which includes, in our opinion, some high-level interpretation of the results. However, if the editor feels strongly, we will remove.

32. L486. Avoid to interpret result in the legend of the Figure 6 L490 and L491-494.

Author response: We prefer to have our figures stand alone, which includes, in our opinion, some high-level interpretation of the results. However, if the editor feels strongly, we will remove.

33. Figure 6. I don’t understand how the index of map resistance can range between -1 and 1, if it calculated from equation L358. The sum should vary between 0 and +inf

Author response: Thank you for catching that. We log-transformed resistance values for better visualization. We have added that clarification to the methods (Lines 379-380): “For display purposes, we log-transformed resistance values when making our resistance maps.”

34. L499. Replace “regions” by “areas”.

Author response: This sentence has been removed.

35. L553-557. I’m not sure to correctly understand the justification for an artifact. Please reformulate.

Author response: This sentence has been removed.

36. L619-621. Do you have an idea of the number of lions killed annually?

Author response: We keep track of all lion mortalities we encounter and are able to verify. The data is too patchy and unreliable to report on.

37. L622-624. Can you develop an example in one sentence?

Author response: We have added (lines 588-591): “Strategies to promote human-lion coexistence in the NCA are being implemented, including the Lion Guardians’ model (Dolrenry et al., 2016) where local pastoralist are employed to mitigate conflict, and monitoring and protect lions, as well as a trial of a conservation incentive payment program based on lion presence (Pekor et al., 2019).”

38. L633. Replace “adaptations” by “adjustments”.

Author response: We have made the suggested change.

**Reviewer #2:**

39. This paper makes great effort to improve our understanding of lion landscape and small-scale space use in the proximity of humans. Such an effort is commendable, and I support the publication of this work. However, I would like to make a couple of suggestions that might improve the manuscript.

The first issue that I would like to see captured in the text is a summarised version of Supplementary information S1. I think it is important that the reader is made aware that for resident males approximately 130 months of resident male data came from 8 animals and that almost half of the data came from only two animals. It is also important to note that 190 months of female data comes from 8 different animals but here more than half of the data are from only two animals. For the nomadic males 117 months of data were obtained from 9 animals but more than half of the data is from only 3 animals. By no means do I want to belittle the amount of effort and resources that went into obtaining the data used, but the question remains on how representative it is for all female, resident, and nomadic lions if for each group most of the data is from two or three individuals. I think this need to be explicitly shown and discussed. I do not think it change the conclusions, but the overall interpretation of results needs to be seen in this context. It will be interesting to see how well model results compare when the 2 or 3 key animals’ data are removed form the data set and the models re-run on the remaining data. A comparison between the full and partial model outcomes might be very revealing, but I do not insist on this for publication of this paper. Just please acknowledge the potential influence the individual preference of 2 or 3 key individuals could potentially have on the results.

Author response: We have added a summary of the S1 Table to the beginning of the results section to make the data limitations clear (Lines 383-388): “We tracked a total of 22 collared lions a total of 437.7 months, including eight females and 15 males. Of the males, six were exclusively nomadic during the time they were monitored, five were exclusively resident with a pride and the remaining four spent some time in both categories (S1 Table). Some animals were more represented in each category than others, with >50% of data for females and resident males coming from two animals, respectively, and >50% of data for nomadic males coming from three animals (S1 Table).”

We have also added an acknowledgement of sample size to the discussion (Lines 563-567): “While our study offers important insights, we note that our sample size is small, with 2-3 key individuals dominating the dataset for each lion category. While small sample sizes are typical of wildlife tracking studies, especially for large carnivores, our conclusions could have been influenced if those individuals were not representative of the rest of the population.”

40. The 2nd issue is the wild prey abundance issue. You recognise that an accurate idea of wild prey availability it is a crucial point of investigation for future studies but then you conclude that is important based on the assumption that EVI and season is correlated to wild prey abundance. This correlation might be true in the west on the short grass plains, but I am not convinced it holds for the entire area. My suggestion is to simply remove any text that try and correlate wet/dry season and EVI to prey abundance or at best state that it might be correlated. I made comments in text about this.

Author response: Thanks for pointing this out. We have tried to temper any discussion of the importance of EVI in terms of prey. In the Discussion, we include this sentence (Lines 570-571): “Although we noted positive selection for greenness (EVI) by resident males during the dry season and nomadic males at night, we cannot conclusively say whether this is related to prey abundance.”

However, EVI is the best proxy we have available and thus we think it remains important to include. We have also cited some more references to support this relationship (lines 254-256): “...enhanced vegetation index (hereafter “EVI”; MODIS) to capture vegetation greenness, proxy for forage quality and quantity (Villamuelas et al. 2016), which tends to influence both wild and domestic herbivore (lion prey) distribution (Zengeya et al 2015).”

41. Another issue is that you deduct that lions do not need to predate on livestock during the wet season but that is in direct contradiction to the findings of a recent paper from the Tsavo area in Kenya. It might be worth having a look at the paper of Olivier, I. R., Tambling, C. J., Müller, L. & Radloff, F. G. T. (2023). Lion (Panthera leo) diet and cattle depredation on the Kuku Group Ranch Pastoralist area in southern Maasailand, Kenya. Wildlife Research, 50(4): 310-324.

Author response: We added data to support this pattern (S1 Fig). We also added further supportive references and explanation (Lines 166-168): “Consequently, we observe relatively more lion-livestock attacks during the dry season (S1 Fig), similar to other studies in the region finding increased attacks and/or risk when availability of native prey is low (Beattie et al., 2020; Olivier et al., 2022; Patterson et al., 2004).”

42. A last point that is probably not necessary to address and might deviate a bit from the focus of the paper is the issue of retaliation and traditional killing of lion. In this and other East African landscapes, a landscape of fear exists for lion, you indirectly acknowledge this, created by the episodic killing of lion and the fashion within which this is done (I am not referring to poisoning but the active pursuit of lion by red clad men with spears). I often wonder how long it will take lion to lose there deep fear of Masai people when this practise is completely abandoned and following on this, how long it will take for lions to start killing people in a much higher frequency that is currently the case. Once that starts to happen this “co-existence” idea might not be all that feasible anymore as losing livestock is very different from losing human lives. I am thus of opinion that a more nuanced approach to retaliation and traditional killings is needed although I also at the same time understand the resentment for the cruelty involved.

Author response: The reviewer makes an interesting point, and although we feel that it is beyond the scope of this manuscript, it is along the lines of a separate paper that we have in review elsewhere, discussing a bit more in detail about the coexistence landscape in Ngorongoro.

43. L. 55-58. Much of this is repetition of the sentence in line 51 & 52

Author response: We have removed the latter part of this sentence to avoid repetition, it now reads (Lines 53-54): “The long-term persistence of large carnivores in fragmented landscapes often depends on the ability of individuals to disperse between isolated subpopulations”

44. L. 76-77. Need a reference for this and perhaps have a look at Olivier et al. 2023. Wildlife Research, 50(4): 310-324, where they show that cattle are killed but not preferred.

Author response: Thank you. This sentence is meant to illustrate why lions may be more vulnerable to persecution than other predators. We have clarified this and added references as suggested by the reviewer (lines 70-72): “However, lions living among pastoralists are especially vulnerable to conflict through retaliatory killings, due to their habits of preying more on high value cattle (*Bos taurus*) than other livestock predators (Jansson, 2023; Kissui, 2008), and lingering by the kill (Kissui, 2008; Patterson et al., 2004) where other livestock predators are more elusive (Kissui, 2008)”

Since Olivier et al. 2023 find that lions prefer natural prey over cattle, we have added that reference to a later sentence (lines 76-79): “However, lions have an established capacity for flexibility, preferring natural prey over livestock where and when available (Olivier et al. 2023) and exhibiting behavioral adaptations to avoid conflict with humans (e.g., becoming more nocturnal (Gaynor et al., 2018; Suraci et al., 2019)), often resulting in improved survival.”

45. L. 81-82. The fear lions have for people due to this traditional killing might very well be the reason that there are actually some co-existence. When lions lose their fear of humans due to no prosecution we might see much more killing of humans and then lions will be removed altogether. I think this aspect needs to be considerer and is conveniently ignored by activists

Author response: A very good point that we also constantly consider while working closely in this area. To add some nuance to this effect, we have added (Lines 74-76): “The killings of lions by hunts and direct spearing, whether for retaliatory or ritual purpose, has undoubtedly shaped a relationship including both fear and respect between people and lions, a necessary trait for their coexistence (Frank et al., 2008; Potratz et al., 2024).”

46. L. 120-122. Why would you expect this?

Author response: We have clarified this (lines 112-117): “Finally, we predict different responses to people during the wet season, depending on the scale. At the landscape-scale, we expect higher avoidance of people due to more abundant and widespread natural prey (i.e., wet season migration), enabling lions to shift their range away from humans. However, at the local-scale (i.e. the immediate surrounding habitat), we expect lower avoidance of people during the wet season, because humans range closer to home and use the area around their settlement less intensely compared to the dry season, presenting a lower risk to lions.”

47. L. 135. Probably time to conform to the new Vachellia and Senegalia genus names???

Author response: We have changed the scientific names here as suggested. (line 129)

48. L. 175. But if these plains are "barren" as described in lien 139 who many cattle can there really be during the dry season.

Author response: The plains do indeed become very “barren” in the dry season, and the biggest constraint is the water availability. The Ndutu area is one very important area that has natural springs that pastoralist families dig out and bucket out water for their livestock. Families from far and wide come to settle temporarily in this wider area during the dry season, herding their livestock far each day for either water or grazing. We have clarified this in the text (lines 164-166): “Thus, lions residing on NCA’s shortgrass plains experience a wet season with abundant natural conflict-free prey, and a dry season with high concentrations of risky prey (i.e., livestock) in the few areas with access to water.” and amended line 133 to read “largely barren”.

49. L. 235. In the table you list this as a proxy for cover. So which is it? It is also a very bold statement to say greenness can act as a proxy for prey density.

Author response: EVI is the best proxy we have available for prey, and thus we think it remains important to include. However, we have clarified what it represents and cited some more references to support this relationship (lines 254-256): “... enhanced vegetation index (hereafter “EVI”; MODIS) to capture vegetation greenness, a proxy for forage quality and quantity (Villamuelas et al. 2016) which tends to influence both wild and domestic herbivore (lion prey) distribution (Zengeya et al 2015).”

To temper any prey-related conclusions based on this imperfect proxy, we have added this to the Discussion (lines 570-571): “Although we noted positive selection for greenness (EVI) by resident males during the dry season and nomadic males at night, we cannot conclusively say whether this is related to prey abundance.”

50. L. 243. How is water availability a human related covariate? Water is essential for water dependent wildlife and to my knowledge there is very few, if any, artificial water points available for wildlife or lion. It is an important variable but not necessarily a human related one?

Author response: Most of these water points are used by both humans (to water livestock) and wildlife daily, thus could relate to avoidance of humans (or attraction to livestock). For this reason, we originally considered it as a human-related variable. We ended up not using it in our models, as it was highly correlated with boma locations, undoubtedly because people settled near these water points. We also removed “Water points” from Table 1, to only include variables used for modeling. This correlation and the eventual removal of this covariate is mentioned in the Methods.

We have added some more explanation for why we regard water points as a human variable (lines 264-266): “The distance to nearest waterpoint is also related to human activity, as water is scarce in the NCA for much of the year, necessitating the use of limited naturally-fed water points for people, livestock and wildlife alike. “

51. L. 411. Selecting or using? It might be that the simply not seek out areas of higher cover and hence by default are found more often in the more open areas?

Author response: This is indeed possible and is why we took a two-scale approach to our analysis. The RSF looks at landscape-scale selection (or use, as pointed out by the reviewer). The SSF looks at the finer scale, given the location of the animal on the landscape, what habitat features do they select for? By taking a step-selection approach, we condition on the last location for each subsequent step, which means that our results more faithfully indicate “selection” than unconditional occurrence/location data.

52. L. 518-519. Reference for this?

Author response: we have added references for this, and made some adjustments in text, that now reads (lines 510-511): “At the local scale, cover was especially important for females, possibly because of their high philopatry, and thus familiarity with the pride’s territory (Elliot et al., 2014a; Pusey and Packer, 1987).“

53. L. 521. I have a real problem with this.

Author response: This has been removed.

54. L. 549. Are there no data on seasonal cattle depredation rates that can support this notion? Olivier 2003 actually found an increase in cattle depredation during the wetter periods

Author response: We added data to support this pattern (S1 Fig). We also further supportive references and explanation (lines 166-168): “Consequently, we observe relatively more lion-livestock attacks during the dry season (S1 Fig), similar to other studies in the region finding increased attacks and/or risk when availability of native prey is low (Beattie et al., 2020; Olivier et al., 2022; Patterson et al., 2004).”

L. 574. Prefer or use?

Author response: See above for details, but since we are analyzing this using step-selection, the case for “selection” or “preference” becomes stronger.

55. L. 595-598. Sentence needs a rewrite

Author response: We have changed this to (lines 560-561): “Our resistance map suggests no barriers to connectivity between lion subpopulations in the NCA at the current levels of human presence and activity.”

56. L. 609. Rewrite

Author response: We have changed this to (lines 574-577): “Second, we used bomas as a proxy for human activity, which might be inadequate to capture human and livestock presence. Exact boma locations shift regularly and especially livestock grazing areas, shift regularly. The latter is particularly important in this context of human-lion conflict, as lions in the NCA tend to attack livestock at pasture (Jansson, 2023).”

57. L. 627-628. You acknowledgment earlier that you do not have the data to support this statement so do not make it.

Author response: This phrase has been removed.
